# Assessment of the microbial interplay during anaerobic co-digestion of wastewater sludge using common components analysis

**DOI:** 10.1371/journal.pone.0232324

**Published:** 2020-05-01

**Authors:** Francesc Puig-Castellví, Laëtitia Cardona, Delphine Jouan-Rimbaud Bouveresse, Christophe B. Y. Cordella, Laurent Mazéas, Douglas N. Rutledge, Olivier Chapleur

**Affiliations:** 1 Université Paris-Saclay, INRAE, AgroParisTech, UMR SayFood, Paris, France; 2 Université Paris-Saclay, INRAE, PRocédés biOtechnologiques au Service de l’Environnement, Antony, France; 3 Groupe “Chimiométrie pour la Caractérisation de Biomarqueurs–C2B”, UMR Physiologie de la Nutrition et du Comportement Alimentaire, AgroParisTech, INRAE, Université Paris-Saclay, Paris, France; Gifu University, JAPAN

## Abstract

Anaerobic digestion (AD) is used to minimize solid waste while producing biogas by the action of microorganisms. To give an insight into the underlying microbial dynamics in anaerobic digesters, we investigated two different AD systems (wastewater sludge mixed with either fish or grass waste). The microbial activity was characterized by 16S RNA sequencing. 16S data is sparse and dispersed, and existent data analysis methods do not take into account this complexity nor the potential microbial interactions. In this line, we proposed a data pre-processing pipeline addressing these issues while not restricting only to the most abundant microorganisms. The data were analyzed by Common Components Analysis (CCA) to decipher the effect of substrate composition on the microorganisms. CCA results hinted the relationships between the microorganisms responding similarly to the AD physicochemical parameters. Thus, in overall, CCA allowed a better understanding of the inter-species interactions within microbial communities.

## Introduction

Anaerobic digestion (AD) is a well-established multistep process for the treatment of organic waste that generates biogas, a gas mixture composed of CH_4_ and CO_2_, by the action of the microorganisms growing in the anaerobic digesters [1]. A critical parameter in AD is the carbon-to-nitrogen (C/N) ratio. AD has been particularly used with wastewater sludge (WAS) obtained from wastewater treatment plants. However, the low C/N ratio of WAS can lead to low digestion efficiency rates and thus, to low biogas production yields [2]. In order to improve WAS conversion to biogas, it can be digested in combination with other substrates richer in carbon. This strategy is also known as anaerobic co-digestion (AcoD).

Substrates (and substrate mixtures) with different nutrient compositions result in different performance outcomes. For example, the use of green waste rich in carbohydrates, such as fruit residues, can lead to the rapid production of volatile fatty acids (VFA), resulting in the acidification of the digester that will inhibit the methanization process [3]. Alternatively to these performance analyses, it is possible to examine the anaerobic digesters from a microbial perspective. Advances in high-throughput 16S DNA and RNA sequencing have allowed to extend our understanding of the microbiome by mapping the microbial diversity of the ecosystems [4,5]. However, while RNA-based sequencing approaches give an insight into the active microorganisms, DNA-based sequencing approaches detect both active and inactive cells. For this reason, DNA-based sequencing approaches are inaccurate for studying the microbial community structure [6].

In the study of anaerobic digestion processes, the analysis of the microbial communities can provide an insightful link between the physicochemical state of these digesters and the growth of the microorganisms [7–9]. Therefore, in waste AcoD, this analysis is not only useful for obtaining a microbial characterization of the digestion process but also to determine the digester conditions (e.g. temperature, pH, % co-substrate) that maximize the growth of the microbial species responsible for the biomass digestion. In this regard, we have investigated from a microbial perspective the AcoD of WAS with garden grass (GG) and fish waste (FW) co-substrates, using the performance experiment presented in Cardona *et al*. (2019) [10].

In studies of ADs monitored by 16S sequencing techniques, microbial populations are often dominated by the same phyla regardless of the reactors’ conditions [7]. For instance, the most recurrent phyla from Bacteria are the *Firmicutes*, the *Bacteroidetes*, and the *Proteobacteria* [7,11]. From Archaea, the classes *Methanobacteria* and *Methanomicrobia* are typically found in Ads [7]. Therefore, in order to identify those microbial populations that specifically respond to the substrate of study, data should be explored at other, lower, taxonomic levels (e.g., class, order, family, genus, or species), and this data exploration should not overlook the less abundant microbial Operational Taxonomic Units (OTUs). However, the data exploration at the lowest taxonomic levels is difficult as some OTUs may still remain unclassified [12].

Datasets containing 16S rRNA sequence counts are usually large, sparse, and over-dispersed [13]. To reduce the complexity of the study, several approaches are commonly pursued: to analyze only those OTUs found above an abundance threshold (i.e., 1% of the total abundance [14]), to analyze Archaea and Bacteria separately [15], and even by carrying out directed analyses on the less abundant populations (i.e., comparing populations of *Methanosarcina* with *Methanosaeta* [16]). However, none of these three approaches allows for a complete data exploration, as they do not take into account the potential relationships (i.e., between Archaea and Bacteria, and between the dominant and the subdominant micro-organisms) in the ADs.

With this aim, in this study we propose a data pre-processing pipeline to reduce the data sparsity and dispersity, allowing the posterior analysis at the lowest taxonomic level of all the microbial community.

The pre-processed data was further examined with the Common Components Analysis (CCA) chemometric method [17,18] to unravel the underlying microbial communities that responded to GG and FW co-substrates. CCA has the advantage over the more commonly used Principal Components Analysis (PCA) in that variables presenting the same effect on the dispersion of the observations will be grouped together in the same Common Component (CC), and that the first CCs will group together the largest number of variables with the greatest effect on a particular dispersion of the individuals [17,18]. Finally, these microbial communities associated with each latent structure (component) were explored from a phylogenetic and ecologic perspective to investigate the foundations of these microbial interactions. Hence, the proposed data analysis pipeline has not only been specifically designed to cope with the particularities of this data type, but also to provide results of significance in ecological terms by revealing potential microbial interactions in the ecosystems studied.

## Material and methods

### Feedstock preparation

Wastewater sludge (WAS) was obtained from an industrial wastewater treatment plant (Valenton, France) operated by Degrémon SAS. Fish waste (FW) was collected from a fish market, and garden grass (GW) was generated by mowing INRAE’s lawn. Organic wastes were then crushed and the resulting solid parts were stored at 4°C for two days.

The inoculum was obtained from a mesophilic full-scale anaerobic digester treating primary sludge at the same wastewater treatment plant. To prepare the inoculum before use, it was left under anaerobic conditions at 35°C for two weeks to digest the remaining residual organic matter.

Degrémon SAS gave permission to collect all sludge sources (WAS and the inoculum) from the wastewater treatment plant.

Chemical characteristics of substrates and inoculum can be consulted in S1 Table.

### Co-digestion experimental set-up

The same experimental set-up from Cardona *et al*. (2019) was used [10]. Nine anaerobic 1 L batch bioreactors bottles were used. Bioreactors were filled with different mixtures of WAS substrate and a co-substrate (FW or GG) prepared in different proportions of grams of chemical oxygen demand (COD). These proportions consisted of 0/100, 25/75, 50/50, 75/25, and 100/0 gCOD WAS/gCOD co-substrate. Next, for every mixture, 12 gCOD were introduced into a reactor, and all bioreactors were inoculated with anaerobic sludge to a final substrate/inoculum ratio of 12 gCOD/1.2 gCOD. All the digesters were complemented with a biochemical potential buffer (International Standard ISO 11734 (1995) [19]) up to a final working volume of 700 mL. The bioreactors were sealed with a screw cap and a rubber septum and headspaces were flushed with N_2_ (purity > 99.99%, Linde gas SA). The reactors were incubated for 4 weeks at 35°C in the dark without agitation and samples were collected weekly using a syringe. Samples were centrifuged at 10,000 g for 10 minutes to separate the pellets from the supernatants. Both pellets and supernatants were snapped frozen in liquid nitrogen. Pellets were kept at -80°C until the RNA extraction, and supernatants were stored at -20ºC until the chemical analysis.

### Microbial structure analysis

For each tested substrate mixture, we only sequenced the 16S rRNA of the 2 samples closest in time (one after and one before) to the maximal methane production capacity (see S1 File). The maximal methane production for WAS was found at day 24, for the WAS-GG co-digestion mixtures between the 14^th^ and the 15^th^ day, and for the WAS-FW co-digestion mixtures between the 22^th^ and the 25^th^ day. So, for digesters containing mixtures of WAS and GG, the sequenced samples were collected at days 14 and 21. For digesters containing mixtures of WAS and FW, the sequenced samples were collected at days 21 and 28. By sampling at these time points, the analysis was driven towards determining the microbial response linked to methanogenesis for the different substrate mixtures used. In order to compare results from GG and FW digesters to those from WAS digesters, samples sequenced from the latter were collected at the 3 screened time-points (days 14, 21, and 28). In total, 19 samples were sequenced. For more detail about the 16S rRNA sequencing analysis [4], see S1 File. The sequencing data have been deposited in the bioproject PRJNA562430, and samples accession numbers go from SAMN12640739 to SAMN12640746, from SAMN12640748 to SAMN12640756, and from SAMN12640758 to SAMN12640759.

### Bioinformatic analysis

The FROGS (Find Rapidly Operational Taxonomic Units with Galaxy Solution) pipeline was used to analyse the 16S rRNA tags reads. FROGS is a galaxy workflow designed to produce an OTU count matrix from high depth sequencing amplicon data [20]. Briefly, the reads comprised between 100 and 500 base pairs (bp) were merged and the resulting dataset was denoised. The reads kept were clustered with Swarm algorithm, and chimera and singleton reads were removed. Finally, the taxonomic affiliation of the remaining reads was determined using Silva132 16S as the reference database, resulting in 1145 different OTUs. The counts for every sample and OTU were arranged into a 19-by-1145 data matrix.

### Overview analysis of the anaerobic digester active populations (traditional analysis)

OTU counts from Archaea and Bacteria were studied separately. For each subset, OTUs that did not exceed 1% of the total abundance in at least one sample were combined and analysed as a single OUT [14]. Then, the relative abundance of the OTUs were plotted in bars using the *phyloseq* R-package [21].

### Proposed data analysis pipeline

OTU abundances from GG and WAS samples collected at days 14 and 21 were grouped into a new matrix, and the same was performed for the OTU abundances from FW and WAS samples collected at days 21 and 28. These resulting two matrices were composed of 10 samples each and they will be referred from now on as the ‘GG dataset’ and the ‘FW dataset’, respectively, since they represent the samples obtained from GG or FW digesters containing mixtures at 5 different proportions of sludge (0%, 25%, 50%, 75%, and 100%).

The microbial diversity for each of the studied datasets was calculated as the number of different OTUs presented in the digesters.

**Fig 1:**
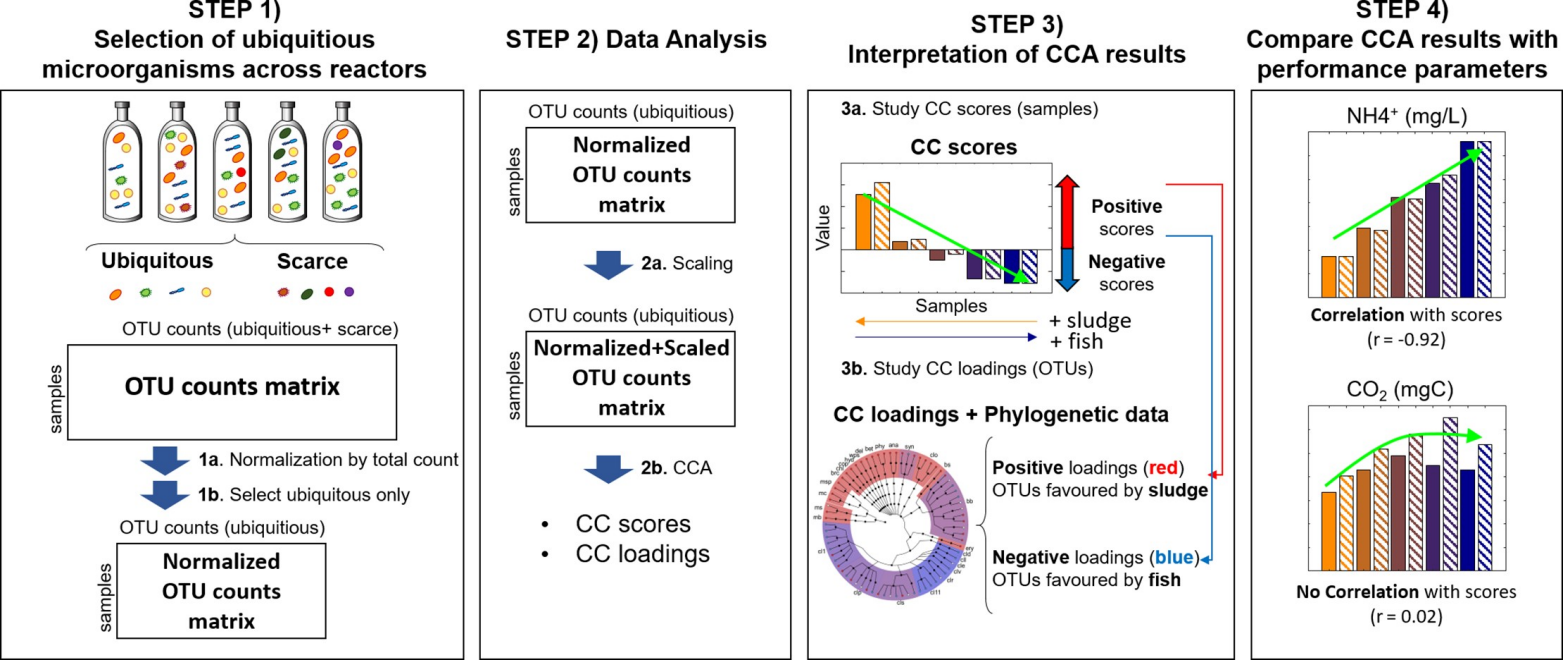
Proposed data analysis pipeline.

Prior to the chemometric analysis, OTU abundances in each sample were divided by their total number of sample reads (Step 1a in **Fig 1**). Moreover, considering the dispersion of the OTUs in the two matrices, OTUs not present in at least 2 samples were discarded and not used in subsequent analyses (Step 1b in **Fig 1**) because their limited presence is proof of a lack of preference for the used substrate. We refer to the set of retained OTUs as ‘ubiquitous’, while the discarded ones are referred as ‘scarce’. Then, the two matrices were Pareto-scaled [22] (Step 2a in **Fig 1**).

Each data matrix was investigated by Common Components Analysis (CCA) (Step 2b in **Fig 1**). CCA is an unsupervised exploratory chemometric method that calculates a series of orthogonal common components (CCs) as linear combinations of the weighted original variables. In these components, variables that present the same dispersion of the observations have strong weightings. This implies that each CC will group together strongly correlated variables, i.e. variables that have the same effect on the dispersion of the observations. As well, the first CCs will group together the largest number of variables with the greatest effect on a particular dispersion of the individuals. As in Principal Components Analysis, each CC has associated scores and loadings vectors. Besides, a vector of weights (called salience) corresponding to the importance of the variables is also obtained iteratively for each CC. For more information regarding CCA, see [17,18].

When CCA is used for the analysis of a 16S dataset, each CC component is representative of an underlying microbial community. CC scores were plotted in bars to examine the relationship between the substrate composition and these microbial communities (Step 3a in **Fig 1**). On the other hand, the phylogeny of the OTUs from these microbial communities was determined from the analysis of their corresponding CC loadings. Representative OTUs for each loading vector were selected after their inspection using S-plots, and they were graphically represented over phylogenetic trees of these OTUs (Step 3b in **Fig 1**) using the GraPhlAn online tool from the Galaxy website (http://huttenhower.sph.harvard.edu/galaxy/).

Finally, to evaluate possible links between the microbial communities and the performance of digesters, the Pearson’s correlation coefficient, *r*, between the CC scores and each of the performance parameters (see S2 Fig) was calculated (Step 4 in **Fig 1**).

## Results

### Overview analysis of the anaerobic digester active populations (traditional analysis)

**Fig 2:**
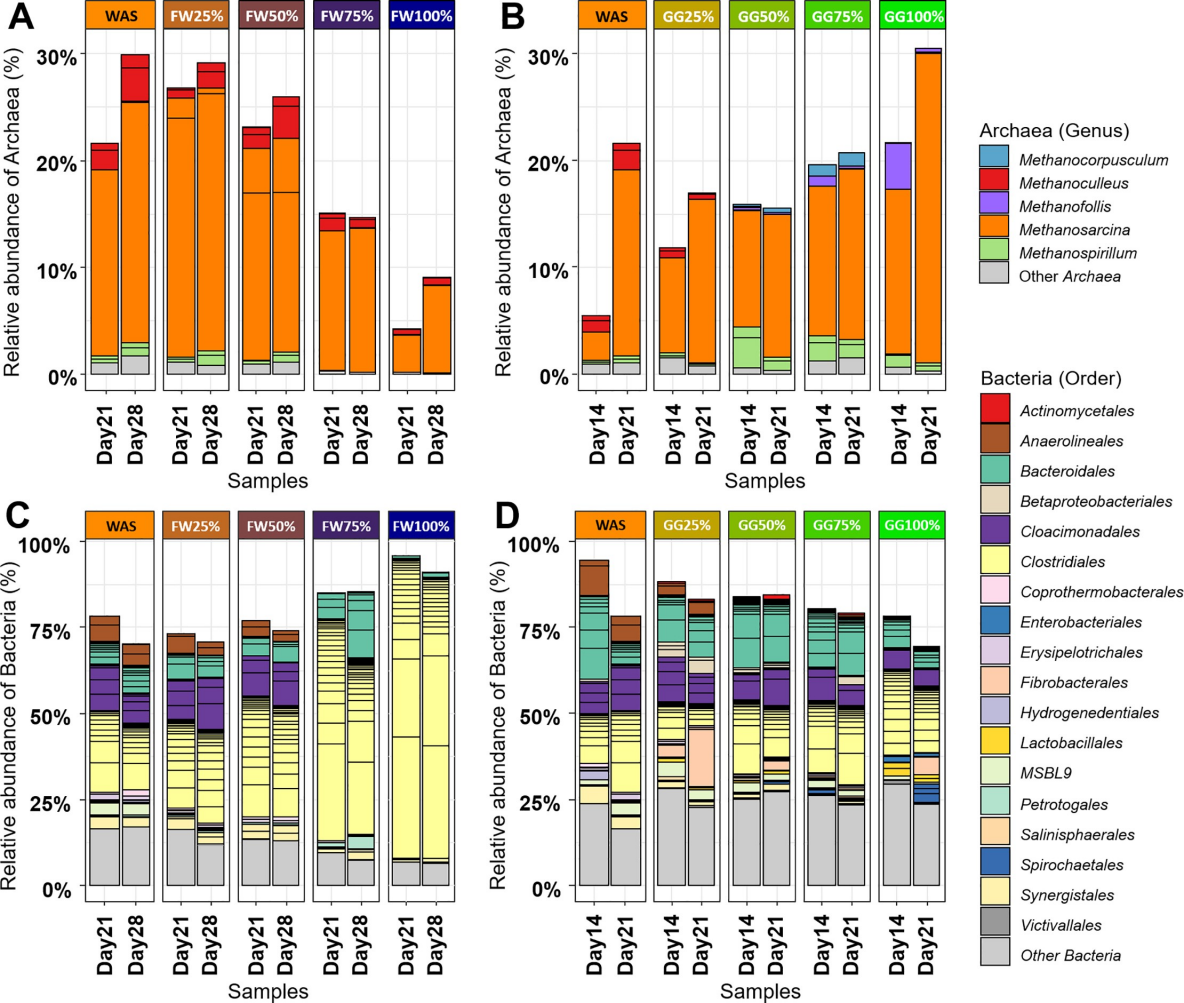
Taxonomic distribution obtained from 16S rRNA sequencing, expressed in percentage of the total number of sequences. **A-B)** Taxonomic distribution of Archaea (coloured by genus) in FW (**A**) and GG (**B**) digesters. **C-D)** Taxonomic distribution of Bacteria (coloured by order) in FW (**C**) and GG (**D**) digesters.

Overall, the microbial diversity consisted of 1145 OTUs. Particular distributions of the species across the digesters can be recognized from the phylogenetic analysis of the OTU counts, as shown in **Fig 2**.

The analysis of the OTU counts revealed that the active population of Archaea was inferior to that of Bacteria in all digesters, ranging between ~4% and ~31% of the total microbial active population (**Fig 2A** and **2B**). Moreover, these differences in relative abundance among digesters were associated with the co-substrates, since the relative abundance of Archaea presented a negative correlation (*r* = -0.87) to FW substrate (**Fig 2A**), and a positive correlation (*r* = 0.69) to GG substrate (**Fig 2B**).

Regarding the Archaea species, its diversity is mainly described by 8 OTUs that presented an abundance superior to 1% in at least one sample. Only 4 of those 8 OTUs were found in FW-digesters, while the number of Archaeal OTUs found in GG-digesters was of 6. In all samples, the most populated genus was *Methanosarcina* (2 OTUs), ranging from ~3% to ~29% of the total microbial population. *Methanosarcina* is a typical genus found in co-digesters of sewage sludge [11]. Other Archaea genera were also detected, with a heterogeneous preference for the different substrates. *Methanoculleus* species (2 OTUs) mainly spanned from 25% GG- to 100% FW-digesters, a *Methanofollis* species (1 OTU) had greater activity in digesters rich in grass, a *Methanocorpusculum* (1 OTU) species was more active in 50% and 75% GG-digesters, and anaerobic digesters with ≥75% fish were found to be hostile AD environments for *Methanospirillum* species (2 OTUs). A similar distribution pattern as for *Methanospirillum* was observed for the Archaea species that accounted for less than 1% of the relative abundance in the digesters (grouped in **Fig 2A** and **2B** as ‘Other Archaea’).

Bacteria were mainly represented by 84 OTUs from 19 different orders that presented an uneven distribution across all screened anaerobic digesters. Bacterial diversity of FW-digesters was composed of 43 OTUs, while the number of Bacterial OTUs found in GG-digesters was of 61. At the order level, GG-digesters (**Fig 2D**) contained a higher diversity than FW-digesters (**Fig 2C**). In the latter, they were clearly dominated by the *Clostridiales* (39 OTUs) order, and *Bacteroidales* (16 OTUs) were also found in all FW-digesters. *Clostridiales* and *Bacteroidales* are known to be two dominant phylogenetic orders adapted to degrading a wide range of substrates [23].

*Synergistales* (2 OTUs) and *Petrotogales* (1 OTU, genera *Defluviitoga*) were specific to the FW substrate, although their activity was negligible in 100% FW-digesters. *Synergistales* have been detected in samples collected in anaerobic digesters ecosystems, and also living as a host in fish [24].

*Anaerolineales* (2 OTUs) and *Cloacimonadales* (4 OTUs) could grow in 0–50% FW-digesters. *Anaerolineales* is a core order found in sewage sludge [25], while *Cloacimonadales* are syntrophic Bacteria that sustain on propionic acid found in anaerobic digesters containing WAS sludge [26].

*Coprothermobacterales* (1 OTU, genera *Coprothermobacter*), *Hydrogenedentiales* (1 OTU) and *Erysipelotrichales* (1 OTU, genera *Turicibacter*) presented their maximal activity for 100% WAS-digesters, whereas they were almost inactive in the rest. *Coprothermobacterales* are hydrogen producers that can establish syntrophic associations with hydrogenotrophic Archaea [27], such as with *Methanoculleus*, or with H_2_-utilizing Bacteria as *Hydrogenedentiales* [28]. On the other hand, *Turicibacter* is typically found in sewage sludge [29].

In GG-digesters (**Fig 2D**), a larger number of bacterial orders were observed than for FW-digesters (**Fig 2C**). Furthermore, most of these orders were specific for a few of the GG-digesters, while the span of the bacterial orders in FW-digesters was less restricted. In other words, the bacterial diversity across GG-digesters was higher than across FW-digesters.

*Bacteroidales*, *Cloacimonadales*, *Clostridiales*, and *MSBL9* (1 OTU) were found in all GG digesters. *Lactobacillales* (2 OTUs), *Enterobacteriales* (1 OTU) and *Spirochaetales* (3 OTUs) were mainly found in 100% GG digesters; *Betaproteobacteriales* (3 OTUs) and *Actinomycetales* (1 OTU) preferred 25%-75% GG-digesters; and *Fibrobacterales* were prominent at the 21^st^ day in 25%, 50%, and 100% GG-digesters. *Lactobacillales* [3], *Enterobacteriales* [30], *Spirochaetales* [31], *Actinomycetales* [32], and *Fibrobacterales* [33] are known to be carbohydrate fermenters.

Finally, in GG digesters, the percentage of bacterial OTUs that did not reach 1% of the total abundance was very high, up to 25% for 100% GG digesters, while for FW-digesters this value was about 10–15%.

### Microbial diversity in anaerobic digesters

The species diversity in the anaerobic digesters was evaluated by studying the dispersion of the OTUs. For this aim, we used an approach that consisted in the classification of all the detected OTUs into two categories (either ‘ubiquitous’ or ‘scarce’) and the posterior exploration of the distribution of ‘ubiquitous’ OTUs across all the samples (Step 1b in **Fig 1**). In this approach, ‘ubiquitous’ OTUs refer to those that were found in > 20% of the samples, while ‘scarce’ OTUs are those not classified as ‘ubiquitous’.

**Fig 3:**
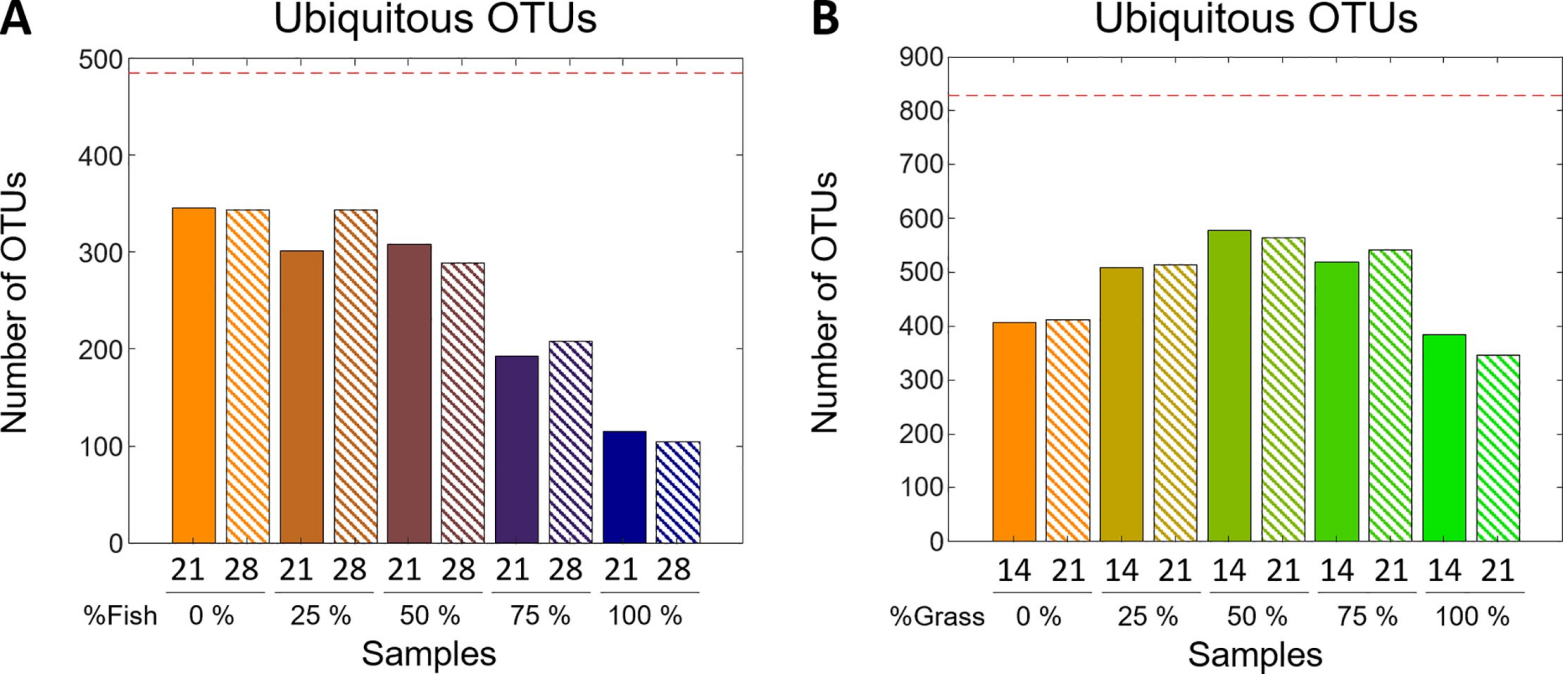
Sample taxonomic diversity. **A)** ‘Ubiquitous’ OTUs in the FW dataset. **B)** ‘Ubiquitous’ OTUs in the GG dataset. For every sample, the collection time and % co-substrate is given as a label on the x-axis. Moreover, % co-substrate is also indicated by a colour-scheme palette ranging from orange to dark blue for 100% WAS to 100% FW, respectively, in the FW dataset; and from orange to green for 100% WAS to 100% GG, respectively, in the GG dataset. Uniform and striped bars correspond to the time-points before and after the maximal methane production for the given co-substrate mixture (see methods). The red dashed lines represent the total number of ubiquitous OTUs.

From the original list of 1145 OTUs, in the FW dataset only 484 OTUs (25 Archaea + 459 Bacteria) could be considered as ‘ubiquitous’, while in the GG dataset 828 OTUs (36 Archaea + 792 Bacteria) were identified as ‘ubiquitous’. We also compared the microbial diversity across the investigated digesters. In the FW dataset, the ‘ubiquitous’ microbial diversity was negatively correlated (*r* = -0.94) to the percentage of fish (a 3.5-fold difference between 0% and 100% FW samples, see **Fig 3A**). On another note, in the GG dataset, the ‘ubiquitous’ microbiome was more diverse when WAS and GG were used in the same proportions. This mixture produced a ~50% increase in the total number of OTUs compared to samples obtained from digesters containing either only WAS or GG (**Fig 3B**).

### Microbial active population dynamics

FW and GG datasets of ‘ubiquitous’ OTUs were explored by CCA (Step 2b in **Fig 1**).

**Fig 4:**
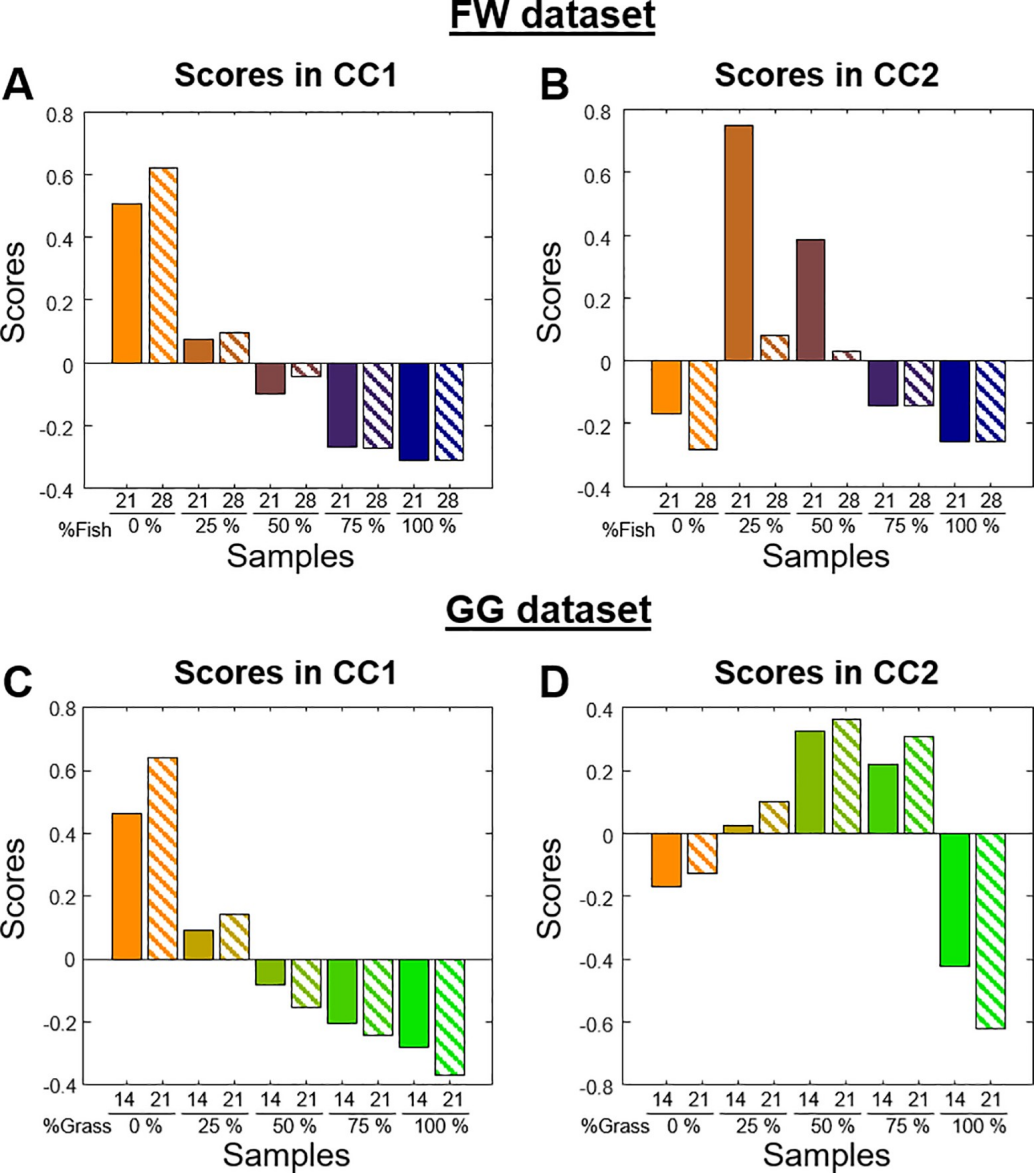
CCA score plots for the FW and GG datasets. **A)** CC1 scores for the FW dataset **B)** CC2 scores for the FW dataset **C)** CC1 scores for the GG dataset **D)** CC2 scores for the GG dataset. The colour-scheme used is the same as in **Fig 3**.

CCA only revealed two interpretable components for each of the two datasets. The CCA scores (**Fig 4**) are descriptive of the species sensitivity to the variations in the substrate composition, regardless of their relative abundance in the anaerobic digesters (Step 3a in **Fig 1**). Both CCA models show similar preference trends for the communities living in the anaerobic digesters. On the one hand, the active microbial population represented by CC1 in the FW dataset includes those OTUs most active in mixtures rich in WAS (by positive scores) or in FW (by negative scores) substrate. On the other hand, CC2 (**Fig 4B**) is representative of those OTUs that were most active in the mixture of WAS and FW substrates (by positive scores), and those most active in the two mono-digestions (100% WAS and 100% FW, by negative scores in **Fig 4B**). An analogous microbial response was interpreted from CC1 and CC2 scores of the GG dataset (**Fig 4C** and **4D**, respectively).

Lastly, in these CCs, the microbial communities were not severely altered over time, except for CC2 from the FW dataset (**Fig 4B**). In this component, there is an important decay of the preference for 25% and 50% FW over time. This response reflects a drastic change in the microbial ecosystem that might be a consequence of the exhaustion of essential nutrients.

With the aim to focus only on the most characteristic OTUs from each population, OTUs with the highest absolute covariance (greater than **±** one standard deviation) with the CC scores were selected employing the S-plots (see S1 Fig). For the FW dataset, 59 OTUs were selected in CC1 and 42 in CC2. In total, 71 OTUs from 18 different orders showed a (positive or negative) response to the FW substrate (see S2 Table). For the GG dataset, 109 OTUs were selected in CC1 and 104 in CC2. In total, for this dataset, 161 OTUs from 36 different orders showed a response to the GG substrate (see S3 Table).

**Fig 5:**
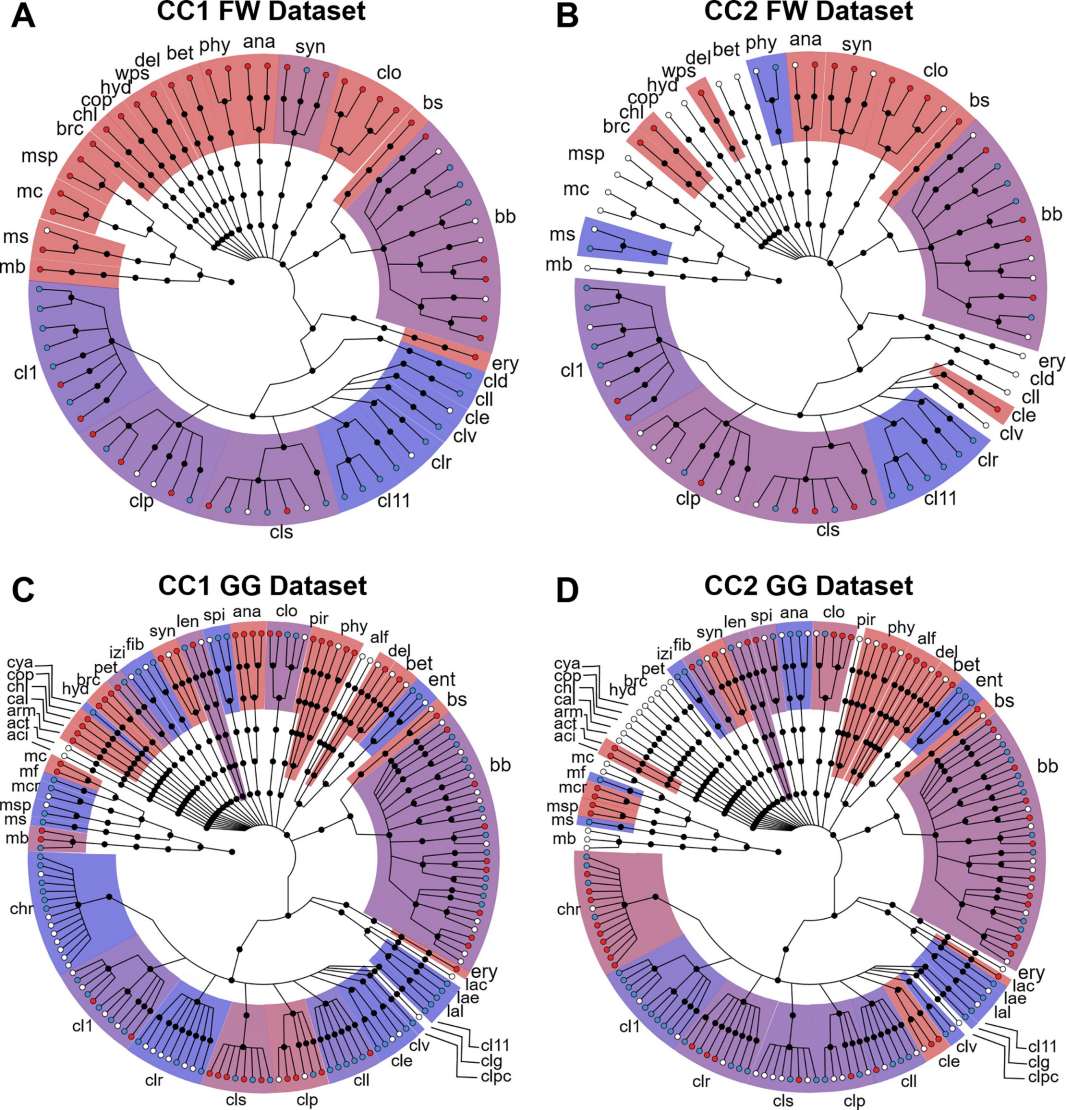
Phylogenetic trees colored according to the CCA loadings from the FW and GG datasets. **A-B**) Phylogenetic trees of the OTUs selected from CC1 loadings (**A**) and CC2 loadings (**B**) for the FW dataset. **C-D**) Phylogenetic trees of the OTUs selected from CC1 loadings (**C**) and CC2 loadings (**D**) for the GG dataset. Positive loadings are coloured in red, negative loadings in cyan, and OTU not selected for the given components were left blank. The shadow colour of every phylogenetic group represents the average loading of the associated OTUs. Labels: aci, *Acidobacteria*; act, *Actinobacteria*; alf, *Alphaproteobacteria*; ana, *Anaerolineae*; arm, *Armatimonadetes*; bb, *Bacteroidales*; bet, *Betaproteobacteriales*; brc, *BRC1*; bs, *Sphingobacteroidales*; cal, *Caldatribacteria*; chl, *Chlamydiae*; chr, *Christensenellaceae*; cld, *DTU014;* cle, *Eubacteriaceae*; clg, *Gracilibacteriaceae*; cll, *Lachnospiraceae*; clo, *Cloacimonetes*; clp, *Peptostreptococcaceae*; clpc, *Peptococcaceae*; clr, *Ruminococcaeceae*; cls, *Syntrophomonadaceae*; clv, *Clostridiales vadinBB60 group*; cl1, *Clostridiaceae* 1; cl11, *Clostridium Family 11*; cop, *Coprothermobacteria*; cya, *Cyanobacteria*; del, *Deltaproteobacteria*; ent, *Enterobacteriales;* ery, *Erysipelotrichales*; fib, fibrobacterales; hyd, *Hydrogenedentiales*; izi, *Izimaplasmatales*; lac, *Carnobacteriaceae*; lae, *Enterococcaceae*; lal, *Leucononostocaceae*; len, *Lentisphaerae*; mb, *Methanobacterium*; mc, *Methanocollis*; mcr, *Methanocorpusculium*; mf, *Methanofollis*; ms, *Methanosarcina*; msp, *Methanospirillum*; pet, *Petrotogales;* phy, *Phycisphaerae*; pir, *Pirellulales*; spi, *Spirochaetales*; syn, *Synergistales*; wps, *WPS-2*.

The OTUs selected from the loadings were plotted in phylogenetic trees (Step 3b in **Fig 1**). These plots are shown in **Fig 5**. For clarity, for every dataset, phylogenetic trees were created from the combined list of selected OTUs in the two components. In this manner, the spatial distribution of the loadings is identical for the two components. The phylogenetic trees descriptive of the FW dataset are shown in **Fig 5A and 5B**, while those corresponding to the GG dataset are shown in **Fig 5C and 5D**.

In these trees, some common trends can be observed. In the phylogenetic tree of the FW dataset colored by CC1 loadings (**Fig 5A**), *Cloacimonadia*, *Anaerolineae*, *Phycisphareae*, and most *Methanomicrobia* were associated with positive loadings (*i*.*e*. preference for WAS substrate as seen in **Fig 4A**). Individual OTUs from *Methanobacteriaceae*, *Syntrophobacterceae*, *Coprothermobacteraceae*, *Hydrogenedensaceae*, *Rhodocyclaceae* (order *Betaproteobacteriales*) also showed positive loadings for this component. On the other hand, most species associated with a negative loadings value (*i*.*e*. preference for FW) were from the *Clostridiales* and to a lesser extent from the *Bacteroidia* classes. When the same phylogenetic tree is colored according to CC2 loadings, positive values (*i*.*e*. preference for the mixture of WAS and FW) were found for *Cloacimonadia* and *Synergistia*, while the negative values (*i*.*e*. preference for 100% WAS and 100% FW) were from the *Clostridia* and *Bacteroidia* classes.

As seen in the overview analysis of the active populations, the microbial communities in digesters containing GG are more complex than those found in digesters containing FW. Although most phylogenetic groups are very heterogeneous, some present a marked substrate preference. For CC1 (**Fig 5C**), *Anaerolineae* and *Phycisphareae* OTUs are associated with positive loadings (*i*.*e*. preference for sludge), while *Methanomicrobia*, and the *Ruminococcaceae* and the *Christensenellaceae* families from the *Clostridiales* class are associated with negative loadings (*i*.*e*. preference for GG). On the other hand, *Alphaproteobacteria* and some *Christensenellaceae* species are more active in the mixture of WAS and GG (positive loadings), while species from the *Enterobacteriales* and the *Clostridiaceae 1* family respond better to the mono-digestion substrates, WAS or GG (negative loadings).

### Link to degradation performance markers

The connection between the microbial populations and the measured degradation performance markers can be easily drawn from the analysis of the CCA scores. This link can be suspected when the evolution of the degradation performance markers across the samples is correlated to the CCA scores (Step 4 in **Fig 1**).

For instance, there is a positive correlation between CC1 and the methanogenic activity (α_app_), indicating that samples containing more organic matter (in this study, FW and GG) presented a higher acetoclastic profile [34]. Specifically, for FW-digesters, Pearson’s correlation between CC1 scores and the α_app_ is of 0.90 (see S3 Fig). For GG-digesters, the correlation is of 0.96.

Other performance indicators can also be connected to the CCA results. For instance, for the FW dataset, NH_4_^+^ is strongly negatively correlated (*r* = -0.92) to CC1, such as most selected *Clostridium* OTUs in the CCA analysis (see S2 Fig). A correlation between *Clostridium* and NH_4_^+^ has been already observed in household biogas digesters [29]. DIC parameter is also highly negatively correlated to CC1 (*r* = -0.89).

For the GG dataset, the parameters that show a high Pearson’s correlation with CC1 were DIC (-0.76), acetate (0.77), biogas (-0.91), and CO_2_ (-0.90) productions (see S2 Fig).

## Discussion

The overview analysis (**Fig 2**) showed that important changes occurred for the active populations of the most dominant OTUs. In addition, across digesters, the difference in the percentage of the bacterial OTUs that did not reach 1% of the total abundance was very high, revealing that in these digesters there exists an important latent microbial population that should not be neglected in the analysis. In agreement with this observation, the high number of ubiquitous OTUs obtained in the analysis of the microbial diversity using the proposed data analysis pipeline (**Fig 3**) hinted that the microbial communities responding to the substrates are significantly more complex than what could be interpreted from the overview analysis.

In the same analysis of the microbial diversity (**Fig 3**), different responses were observed for the two sets of ADs. On one hand, in FW digesters, the number of ubiquitous OTUs inversely correlated to FW implies that this substrate imposed a strong selective pressure (**Fig 3A**). On the other hand, in GG digesters, the synergic effect of the two substrates observed on the higher diversity of 50% GG-digesters (**Fig 3B**) may be the consequence of having combined two complementary substrates, resulting in a better macro- and micro-nutrient equilibrium, or due to the dilution of inhibitory or toxic compounds present in the mono-digestion substrates.[35] These two reported alterations in the microbial diversity were not spotted in the traditional analysis, pointing out that most of the affected species were sub-dominant.

Regarding the two CCA analyses (**Fig 4**), in broad terms, similar results were obtained. In them, a gradual shift of the microbial community due to substrate composition was observed, and the observed differences over time were smaller than those between reactors. Nevertheless, CC scores were easier to interpret as each of the CCs represents those OTUs that responded similarly to the substrate, while to retrieve the same information from **Fig 2** required the re-analysis of the OTUs one by one.

When the results from the traditional analysis (**Fig 2**) are compared with those from the proposed data analysis pipeline (**Figs 3–5**), four important remarks can be given.

First, it can be seen that the analysis of the 16S rRNA data by CCA combined with the phylogenetic representation of the loadings allowed to analyze simultaneously the archaeal and the bacterial population. In the traditional analysis of these type of data, Archaea and Bacteria are typically analyzed separately due to the important differences in relative abundance.^4^ Therefore, the methodology proposed here provides an advantage in the interpretation, since it deepens our understanding of the interaction among all these different species living in the anaerobic digesters.

Second, more OTUs (and more orders) were selected in the proposed method than in the traditional analysis. Since the method does not depend on the measured abundances, but on the relative measured response across the measured samples, it is possible to detect some latent species that were not highlighted in the analysis of the taxonomic distribution of **Fig 2**. The analysis in **Fig 2** only made it possible to observe 92 OTUs from 19 bacterial orders and 5 archaeal genera, while CCA selected a total of 182 OTUs (in at least one of the datasets) from [35] bacterial orders and 6 archaeal genera. Examples of the relevant OTUs according to the CCA analysis but not evaluated in **Fig 2** due to their abundance include *Alphaproteobacteria* (alf in **Fig 5**), *Deltaproteobacteria* (del), *Izimaplasmatales* (izi), *Lentisphareae* (len), and *Methanobacterium* (mb). Thus, in overall, the developed methodology avoids overlooking the less abundant species, while at the same time resulting on a better understanding of the analyzed microbial populations.

Third, the phylogenetic representation (**Fig 5**) allows investigating the selected OTUs at all taxonomic levels at once, while this is not possible from the traditional analysis (**Fig 2**).

And fourth, all selected OTUs in the CCA responded to the substrate, which allows for an easier and more direct interpretation of the active population dynamics associated with the anaerobic co-digestion of sludge.

Furthermore, the most notable information that can be retrieved from the CCA analyses is that OTUs from the same component present a similar substrate preference, pointing out that they may have established a syntrophic relationship in the digesters. For example, from the analysis of the CC1 loadings from the FW dataset (**Fig 5A**), it can be presumed that *Methanomicrobia* species may grow on the CO_2_ released from other microbial species associated with similar loading values, such as *Anaerolineae* or *Phycisphareae*. Since these species are favoured by WAS, *Methanomicrobia* species are indirectly favoured as well by the same substrate. However, when the CC1 loadings from the GG dataset (**Fig 5C**) are investigated, it is observed that *Methanomicrobia* responds positively to GG. This preference can be interpreted as these Archaea grow on CO_2_ mainly obtained from the fermentation of carbohydrates by the cellulolytic *Lactobacillales*, *Ruminococcaceae* and *Christensenellaceae*, among other bacteria [36–38].

Finally, the reduction of the data complexity into a set of scores and loadings enables to investigate, by simple means, the possible connections between the microbial populations and the measured degradation performance markers (as indicated in Step 4 in **Fig 1**).

For example, the observed correlation between CC1 and the methanogenic acetoclastic activity may be the product of a *Methanosarcina* species (OTU 6 in S2 Table), which showed preference for the fish substrate. *Methanosarcina* grows predominantly on acetoclastic methanogenesis when it is provided by fats, oil, and grease [16] (all present in fish waste). On the other hand, the more prominent hydrogenotrophic profile in 100% WAS-digesters may be derived from the presence of *Coprothermobacterales*, since they are important hydrogen producers [27].

Moreover, the observed correlation of both NH_4_^+^ and DIC to the amount of FW substrate (CC1) denotes an alkalization of the pH in these digesters. NH_4_^+^ and alkali pH can inhibit microbial growth [39]. Thus, in FW-digesters, NH_4_^+^ production during fish degradation may have caused the inhibition of several microorganisms, allowing the growth of NH_4_^+^-resistant microorganisms such as *Clostridium*.

For the GG dataset, the parameters DIC, acetate, biogas, and CO_2_ productions were correlated to CC1. These four parameters can be linked to the preference of carbohydrate fermenter Bacteria and acetoclastic Archaea to grass waste substrate. This preference led to the increase of CO_2_ levels (by Bacteria), and the increase of biogas mediated by the consumption of acetate levels (by Archaea). Moreover, the increase of CO_2_(g) production, in turn, could elevate the levels of dissolved CO_2_, which would explain the increase in the DIC measurements.

## Conclusions

The effect of substrate composition on microbial communities from AcoD can be investigated by CCA, which grouped together in separate Components the microorganisms that respond similarly to the substrates. CCA analysis showed that changes in the community structure due to the substrate occurred for microorganisms present at all relative abundance levels. Moreover, the CCA models revealed that dozens of species responded selectively to the co-substrates, and some of these substrate preferences of the microorganisms were largely class- or order-specific.

The analysis of CCA scores together with the degradation performance markers allowed to establish connections between these markers and the latent microbial communities described by these Components. Specific to FS AcoD, FW caused an important decrease in the microbial population due to NH_4_^+^ being produced during fish degradation. Conversely, in GS AcoD, GG enhanced microbial diversity. In addition, the inclusion of either of the two co-substrates produced a switch in the methanogenic metabolism, from hydrogenotrophic (in WAS) to acetoclastic (in FW or GG), mediated mainly by *Methanosarcina* species.

In all, the developed pipeline avoids overlooking the less abundant species, while at the same time resulting on a better understanding of the analyzed microbial populations. Since this methodology allows for a full understanding of any 16S RNA sequencing dataset, it has the potential to unravel the dynamics of other microbial ecosystems important for the agricultural and food chemistry field, such as in food fermentation processes [8] or to study the gut microbiota [9,40].

## Supporting information

S1 Fig S-plots. (TIF)

S2 Fig Effect of the co-substrate on AD performance. (TIF)

S3 Fig Effect of the co-substrate on the methanogenic pathway. (TIF)

S4 Fig Phylogenetic trees colored according to the CCA loadings from the FW and GG dataset. (TIF)

S1 File Supplementary methods. (DOCX)

S1 Table Characteristics of substrates and inoculum. (DOCX)

S2 Table Taxonomic affiliation for the selected OTUs in FW dataset. (DOCX)

S3 Table Taxonomic affiliation for the selected OTUs in GG dataset. (DOCX)

S1 Data (PDF)

## Abbreviations

AcoD: anaerobic co-digestion
AD: anaerobic digestion
CC: Common Component
CCA: Common Components Analysis
COD: Chemical Oxygen Demand
C/N: carbon-to-nitrogen ratio
DIC: Dissolved Inorganic Carbon
FW: fish waste
GG: garden grass
OUT: Operational Taxonomic Units
VFA: volatile fatty acids
WAS: Wastewater sludge

## References

[1] Fermoso FG, Serrano A, Alonso-Fariñas, Fernández-Bolaños J, Borja R, Rodríguez-Gutiérrez G. Valuable Compound Extraction, Anaerobic Digestion, and Composting: A Leading Biorefinery Approach for Agricultural Wastes. *J*. *Agric*. *Food Chem*. 2018, 66 (32), 8451–8468. 10.1021/acs.jafc.8b02667 30010339

[2] Li Y, Park SY, Zhu J. Solid-State Anaerobic Digestion for Methane Production from Organic Waste. *Renew*. *Sustain*. *Energy Rev*. 2011, 15 (1), 821–826. 10.1016/J.RSER.2010.07.042.

[3] Garcia-Peña EI, Parameswaran P, Kang DW, Canul-Chan M, Krajmalnik-Brown R. Anaerobic Digestion and Co-Digestion Processes of Vegetable and Fruit Residues: Process and Microbial Ecology. *Bioresour*. *Technol*. 2011, 102 (20), 9447–9455. 10.1016/j.biortech.2011.07.068 21865034

[4] Madigou C, Lê Cao KA, Bureau C, Mazéas L, Déjean S, Chapleur O. Ecological Consequences of Abrupt Temperature Changes in Anaerobic Digesters. *Chem*. *Eng*. *J*. 2019, 361, 266–277. 10.1016/J.CEJ.2018.12.003.

[5] Amha YM, Sinha P, Lagman J, Gregori M, Smith AL. Elucidating Microbial Community Adaptation to Anaerobic Co-Digestion of Fats, Oils, and Grease and Food Waste. *Water Res*. 2017, 123, 277–289. 10.1016/j.watres.2017.06.065 28672212

[6] Blazewicz SJ, Barnard RL, Daly RA, Firestone MK. Evaluating RRNA as an Indicator of Microbial Activity in Environmental Communities: Limitations and Uses. *ISME J*. 2013, 7 (11), 2061–2068. 10.1038/ismej.2013.102 23823491 PMC3806256

[7] Wang NX, Lu XY, Tsang YF, Mao Y, Tsang CW, Yueng VA. A Comprehensive Review of Anaerobic Digestion of Organic Solid Wastes in Relation to Microbial Community and Enhancement Process. *J*. *Sci*. *Food Agric*. 2019, 99 (2), 507–516. 10.1002/jsfa.9315 30144051

[8] Duan S, Hu X, Li M, Miao J, Du J, Wu R. Composition and Metabolic Activities of the Bacterial Community in Shrimp Sauce at the Flavor-Forming Stage of Fermentation As Revealed by Metatranscriptome and 16S RRNA Gene Sequencings. *J*. *Agric*. *Food Chem*. 2016, 64 (12), 2591–2603. 10.1021/acs.jafc.5b05826 26978261

[9] Ntemiri A, Ribière C, Stanton C, Ross RP, O’Connor EM, O’Toole PW. Retention of Microbiota Diversity by Lactose-Free Milk in a Mouse Model of Elderly Gut Microbiota. *J*. *Agric*. *Food Chem*. 2019, 67 (7), 2098–2112. 10.1021/acs.jafc.8b06414 30665298

[10] Cardona L, Levrard C, Guenne A, Chapleur O, Mazéas L. Co-Digestion of Wastewater Sludge: Choosing the Optimal Blend. *Waste Manag*. 2019, 87, 772–781. 10.1016/j.wasman.2019.03.016 31109580

[11] Abendroth C, Vilanova C, Günther T, Luschnig O, Porcar M. Eubacteria and Archaea Communities in Seven Mesophile Anaerobic Digester Plants in Germany. *Biotechnol*. *Biofuels* 2015, 8 (1), 87 10.1186/s13068-015-0271-6. 26097504 PMC4474353

[12] Treu L, Kougias PG, Campanaro S, Bassani I, Angelidaki I. Deeper Insight into the Structure of the Anaerobic Digestion Microbial Community; the Biogas Microbiome Database Is Expanded with 157 New Genomes. *Bioresour*. *Technol*. 2016, 216, 260–266. 10.1016/j.biortech.2016.05.081 27243603

[13] Li H. Microbiome, Metagenomics, and High-Dimensional Compositional Data Analysis. *Annu*. *Rev*. *Stat*. *Its Appl*. 2015, 2 (1), 73–94. 10.1146/annurev-statistics-010814-020351.

[14] Kuczynski J, Stombaugh J, Walters WA, González A, Caporaso JG, Knight R. Using QIIME to Analyze 16S RRNA Gene Sequences from Microbial Communities. *Curr*. *Protoc*. *Bioinforma*. 2011, *Chapter 10*, Unit 10.7. 10.1002/0471250953.bi1007s36. PMC3249058 22161565

[15] Kirkegaard RH, McIlroy SJ, Kristensen JM, Nierychlo M, Karst SM, Dueholm MS, et al The Impact of Immigration on Microbial Community Composition in Full-Scale Anaerobic Digesters. *Sci*. *Rep*. 2017, 7 (1), 9343 10.1038/s41598-017-09303-0 28839166 PMC5571154

[16] Kurade MB, Saha S, Salama ES, Patil SM, Govindwar SP, Jeon BH. Acetoclastic Methanogenesis Led by Methanosarcina in Anaerobic Co-Digestion of Fats, Oil and Grease for Enhanced Production of Methane. *Bioresour*. *Technol*. 2019, 272, 351–359. 10.1016/j.biortech.2018.10.047 30384210

[17] Bouhlel J, Jouan-Rimbaud Bouveresse D, Abouelkaram S, Baéza E, Jondreville C, Travel A, et al Comparison of Common Components Analysis with Principal Components Analysis and Independent Components Analysis: Application to SPME-GC-MS Volatolomic Signatures. *Talanta* 2018, 178, 854–863. 10.1016/j.talanta.2017.10.025 29136906

[18] Rutledge DN. Comparison of Principal Components Analysis, Independent Components Analysis and Common Components Analysis. *J*. *Anal*. *Test*. 2018, 2 (3), 235–248. 10.1007/s41664-018-0065-5.

[19] International Organization for Standardization, Geneva, S. ISO 11734:1995—Water quality—Evaluation of the anaerobic biodegradability of organic compounds in digested sludge—Method by measurement of the biogas production https://www.iso.org/standard/19656.html (accessed Jan 29, 2019).

[20] Escudié F, Auer L, Bernard M, Mariadassou M, Cauquil L, Vidal K, et al FROGS: Find, Rapidly, OTUs with Galaxy Solution. *Bioinformatics* 2018, 34 (8), 1287–1294. 10.1093/bioinformatics/btx791 29228191

[21] McMurdie PJ, Holmes S. Phyloseq: An R Package for Reproducible Interactive Analysis and Graphics of Microbiome Census Data. *PLoS One* 2013, 8 (4), e61217 10.1371/journal.pone.0061217 23630581 PMC3632530

[22] van den Berg RA, Hoefsloot HC, Westerhuis JA, Smilde AK, van der Werf MJ. Centering, Scaling, and Transformations: Improving the Biological Information Content of Metabolomics Data. *BMC Genomics* 2006, 7 (1), 142 10.1186/1471-2164-7-142. 16762068 PMC1534033

[23] Panichnumsin P, Ahring B, Nopharatana A, Chaiprasert P. Microbial Community Structure and Performance of an Anaerobic Reactor Digesting Cassava Pulp and Pig Manure. *Water Sci*. *Technol*. 2012, 66 (7), 1590–1600. 10.2166/wst.2012.358 22864448

[24] Godon JJ, Moriniere J, Moletta M, Gaillac M, Bru V, Delgenes JP. Rarity Associated with Specific Ecological Niches in the Bacterial World: The “Synergistes” Example. *Environ*. *Microbiol*. 2005, 7 (2), 213–224. 10.1111/j.1462-2920.2004.00693.x 15658988

[25] Rivière D, Desvignes V, Pelletier E, Chaussonnerie S, Guermazi S, Weissenbach J, et al Towards the Definition of a Core of Microorganisms Involved in Anaerobic Digestion of Sludge. *ISME J*. 2009, 3 (6), 700–714. 10.1038/ismej.2009.2 19242531

[26] Calusinska M, Goux X, Fossépré M, Muller EEL, Wilmes P, Delfosse P. A Year of Monitoring 20 Mesophilic Full-Scale Bioreactors Reveals the Existence of Stable but Different Core Microbiomes in Bio-Waste and Wastewater Anaerobic Digestion Systems. *Biotechnol*. *Biofuels* 2018, 11 (1), 196 10.1186/s13068-018-1195-8. 30038663 PMC6052691

[27] Gagliano MC, Braguglia CM, Petruccioli M, Rossetti S. Ecology and Biotechnological Potential of the Thermophilic Fermentative Coprothermobacter Spp. *FEMS Microbiol*. *Ecol*. 2015, 91 (5). 10.1093/femsec/fiv018. 25764466

[28] Nobu MK, Narihiro T, Rinke C, Kamagata Y, Tringe SG, Woyke T, et al Microbial Dark Matter Ecogenomics Reveals Complex Synergistic Networks in a Methanogenic Bioreactor. *ISME J*. 2015, 9 (8), 1710–1722. 10.1038/ismej.2014.256 25615435 PMC4511927

[29] Rui J, Li J, Zhang S, Yan X, Wang Y, Li X. The Core Populations and Co-Occurrence Patterns of Prokaryotic Communities in Household Biogas Digesters. *Biotechnol*. *Biofuels* 2015, 8 (1), 158 10.1186/s13068-015-0339-3. 26413157 PMC4582640

[30] Hartemink R, Van Laere KMJ, Rombouts FM. Growth of Enterobacteria on Fructo-Oligosaccharides. *J*. *Appl*. *Microbiol*. 1997, 83 (3), 367–374. 10.1046/j.1365-2672.1997.00239.x 9351217

[31] Harwood CS, Canale-Parola E. Branched-Chain Amino Acid Fermentation by a Marine Spirochete: Strategy for Starvation Survival. *J*. *Bacteriol*. 1981, 148 (1), 109–116. 7287622 10.1128/jb.148.1.109-116.1981 PMC216172

[32] Saini A, Aggarwal NK, Sharma A, Yadav A. Actinomycetes: A Source of Lignocellulolytic Enzymes. *Enzyme Res*. 2015, 2015, 1–15. 10.1155/2015/279381. PMC4697097 26793393

[33] Ransom-Jones E, Jones DL, McCarthy AJ, McDonald JE. The Fibrobacteres: An Important Phylum of Cellulose-Degrading Bacteria. *Microb*. *Ecol*. 2012, 63 (2), 267–281. 10.1007/s00248-011-9998-1 22213055

[34] Qu X, Mazéas L, Vavilin VA, Epissard J, Lemunier M, Mouchel JM, et al Combined Monitoring of Changes in Δ13CH4 and Archaeal Community Structure during Mesophilic Methanization of Municipal Solid Waste. *FEMS Microbiol*. *Ecol*. 2009, 68 (2), 236–245. 10.1111/j.1574-6941.2009.00661.x 19302549

[35] Mata-Alvarez J, Dosta J, Romero-Güiza MS, Fonoll X, Peces M, Astals S. A Critical Review on Anaerobic Co-Digestion Achievements between 2010 and 2013. *Renew*. *Sustain*. *Energy Rev*. 2014, 36, 412–427. 10.1016/J.RSER.2014.04.039.

[36] Thomas S. Production of Lactic Acid from Pulp Mill Solid Waste and Xylose Using Lactobacillus Delbrueckii (NRRL B445). *Appl*. *Biochem*. *Biotechnol*. 2000, 84–86 (1–9), 455–468. 10.1385/ABAB:84-86:1-9:455. 10849812

[37] Pavlostathis SG, Miller TL, Wolin MJ. Fermentation of Insoluble Cellulose by Continuous Cultures of Ruminococcus Albus. *Appl*. *Environ*. *Microbiol*. 1988, 54 (11), 2655–2659. 16347769 10.1128/aem.54.11.2655-2659.1988 PMC204351

[38] Li W, Khalid H, Zhu Z, Zhang R, Liu G, Chen C, et al Methane Production through Anaerobic Digestion: Participation and Digestion Characteristics of Cellulose, Hemicellulose and Lignin. *Appl*. *Energy* 2018, 226, 1219–1228. 10.1016/J.APENERGY.2018.05.055.

[39] Eldem NÖ, Ozturk I, Soyer E, Callı B, Akgiray Ö. Ammonia and PH Inhibition in Anaerobic Treatment of Wastewaters, Part I: Experimental. *J*. *Environ*. *Sci*. *Heal*. *Part A* 2004, 39 (9), 2405–2420. 10.1081/ESE-200026297. 15478932

[40] Kitts WD, Underkofler L. A. Digestion by Rumen Microorganisms, Hydrolytic Products of Cellulose and the Cellulolytic Enzymes. *J*. *Agric*. *Food Chem*. 1954, 2 (12), 639–645. 10.1021/jf60032a007.

