# Supplementary figures and images for: Assessment of the microbial interplay during anaerobic co-digestion of wastewater sludge using common components analysis
Source: PLoS One. 2020 May 1;15(5):e0232324. doi: 10.1371/journal.pone.0232324 (PMC7194399; doi:10.1371/journal.pone.0232324)

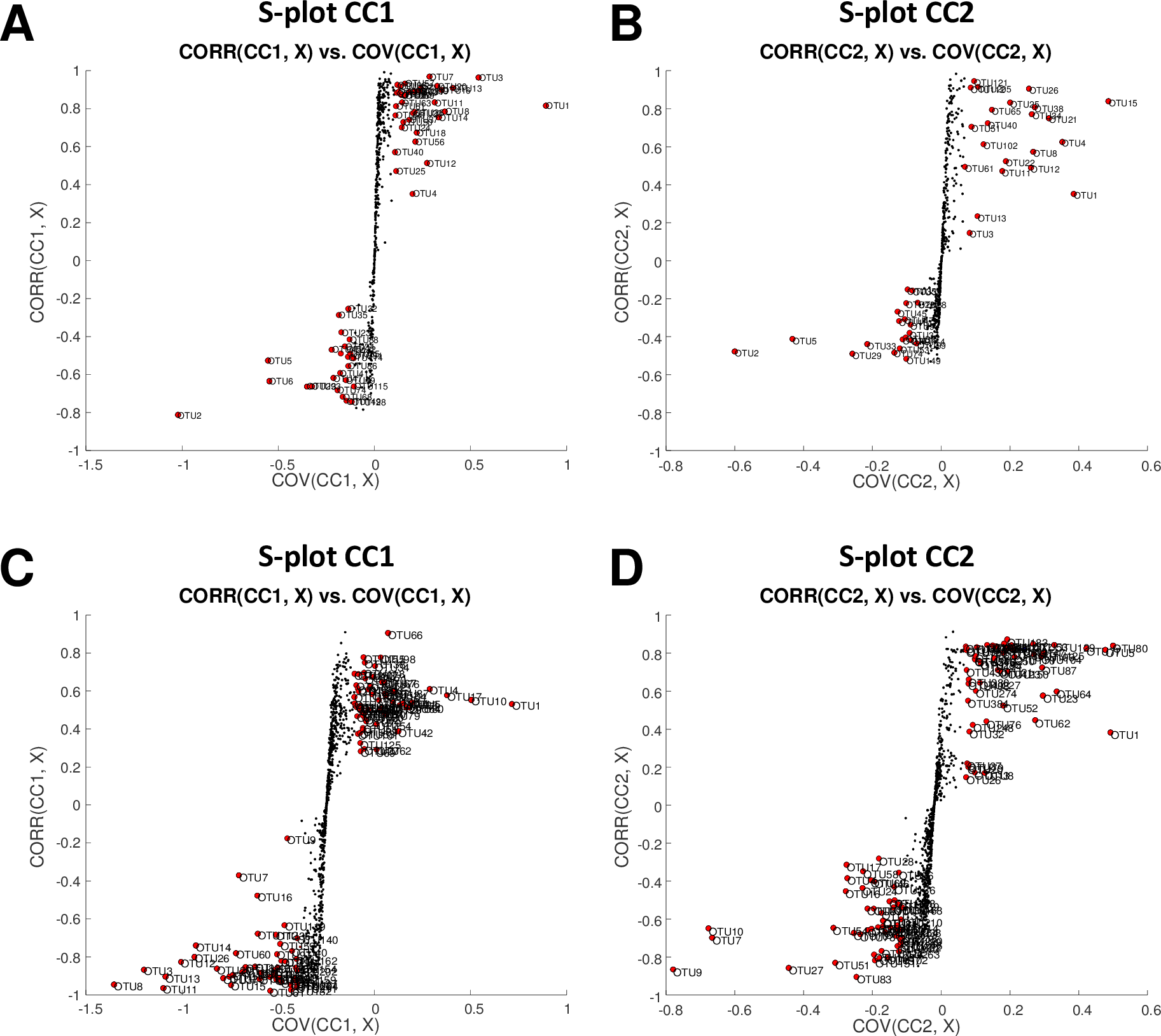

Supplement: S1 Fig — (TIF) [file pone.0232324.s001.tif]

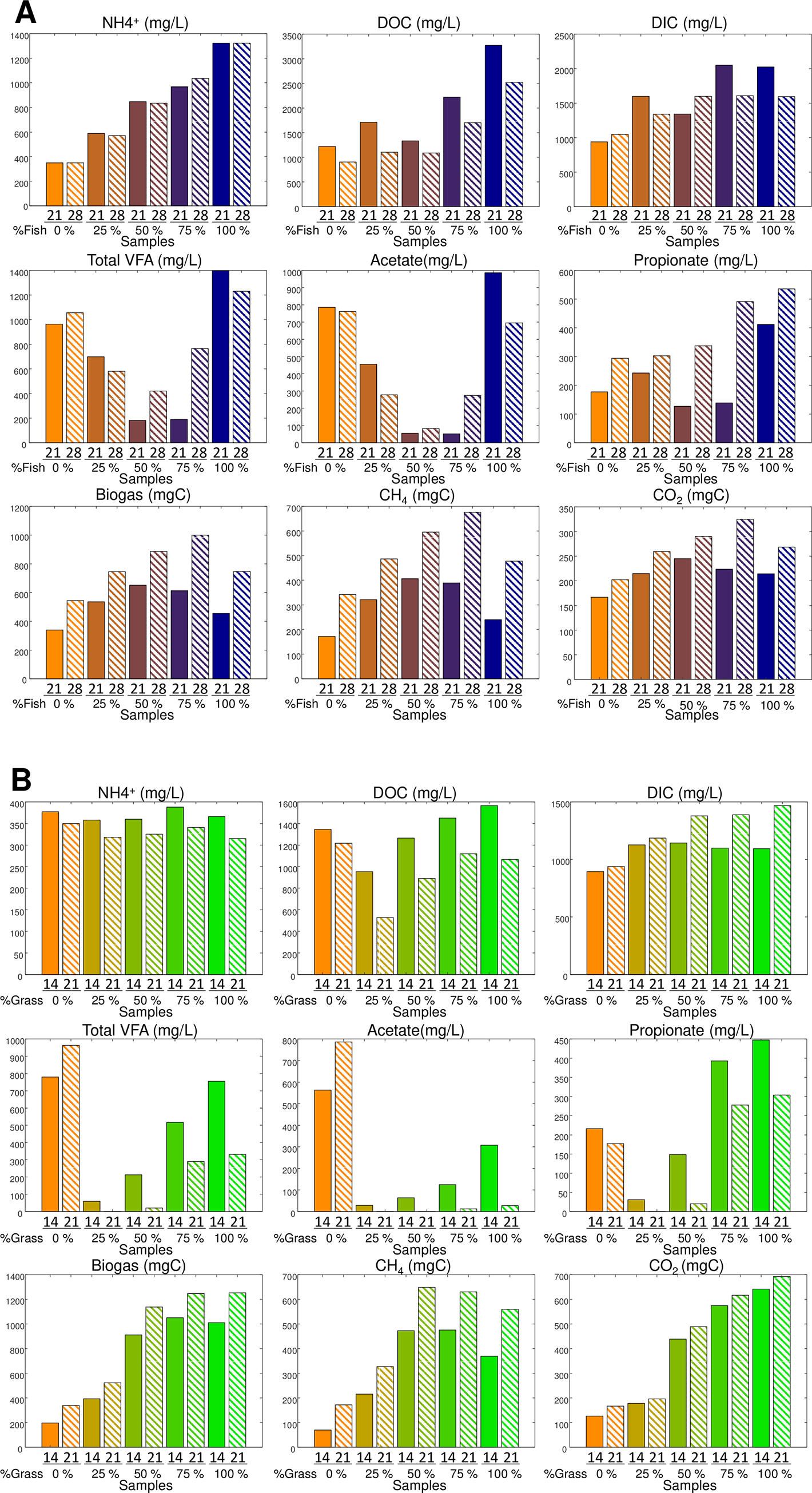

Supplement: S2 Fig — (TIF) [file pone.0232324.s002.tif]

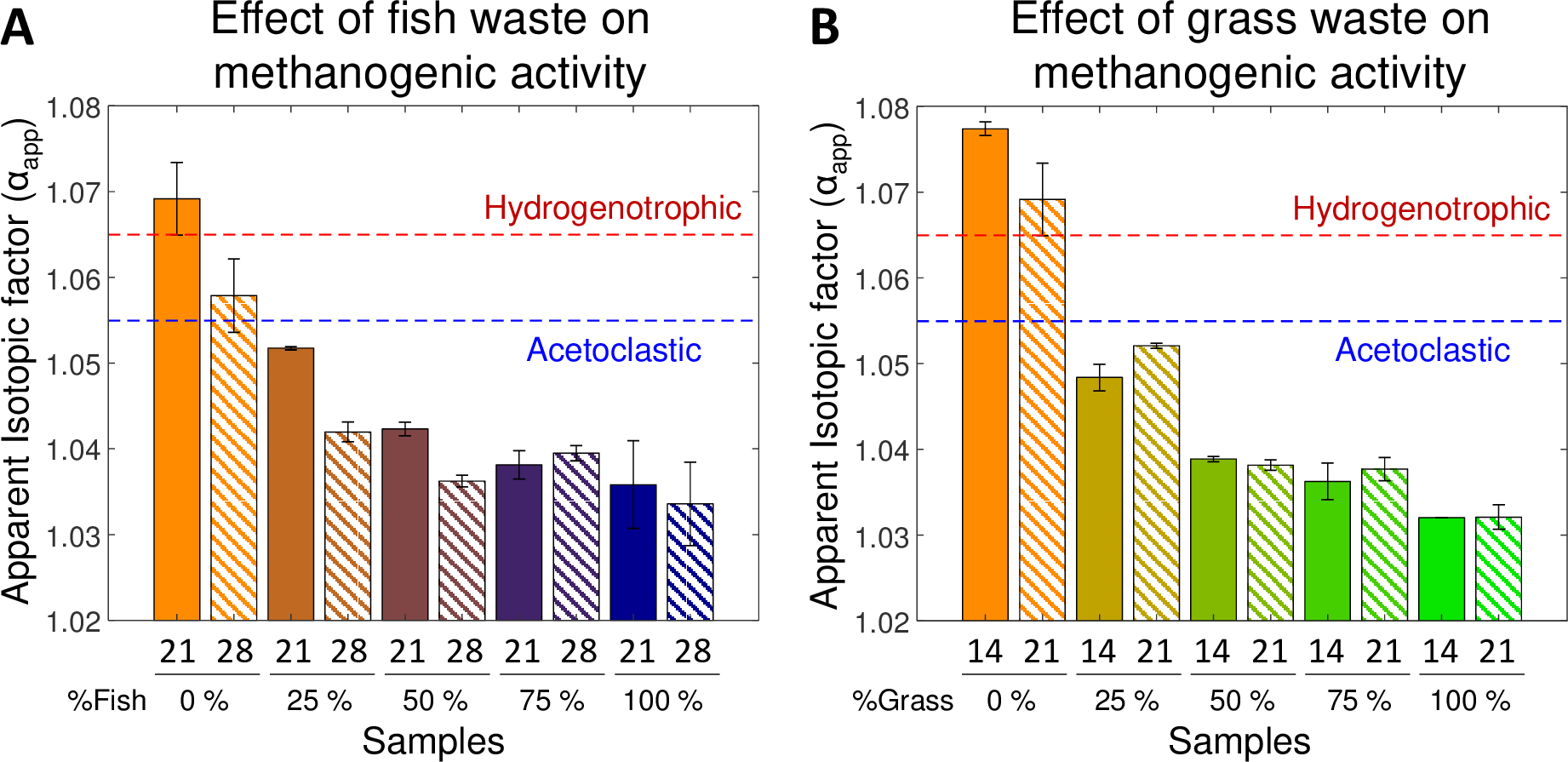

Supplement: S3 Fig — (TIF) [file pone.0232324.s003.tif]

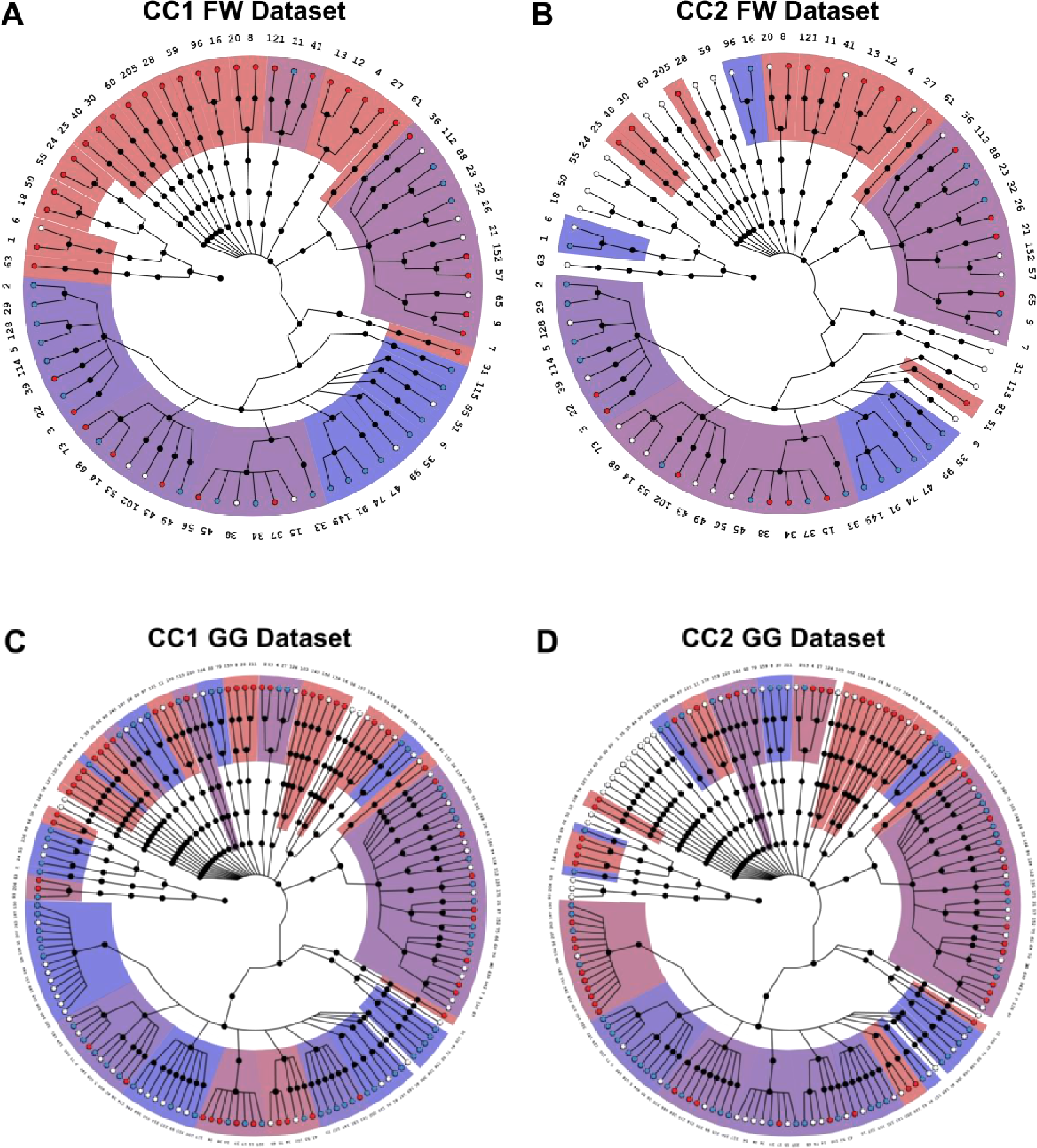

Supplement: S4 Fig — (TIF) [file pone.0232324.s004.tif]
