# Supplementary material for: Assessment of the microbial interplay during anaerobic co-digestion of wastewater sludge using common components analysis
Source: PLoS One. 2020 May 1;15(5):e0232324. doi: 10.1371/journal.pone.0232324 (PMC7194399; doi:10.1371/journal.pone.0232324)
Supplement: S1 File — (DOCX) [file pone.0232324.s005.docx]

PLOS ONE

**Supplementary methods**

**Gas production measurements**

Biogas accumulation in the headspace was measured daily using a differential manometer (Digitron 2082P), and methane production was determined by micro gas chromatography as previously described by Chapleur [1]. These data were used to calculate methane production at standard temperature and pressure.

The isotopic compositions of CH_4_ and CO_2_ (δ^13^CH_4_ and δ^13^CO_2_) were measured to establish whether methane was produced through an acetoclastic or a hydrogenotrophic methanogenesis process. Gas was sampled into 7-mL vacuumed serum tubes, and analysed in a Trace Gas Chromatograph Ultra (Thermo Scientific) attached to a Delta V Plus isotope ratio mass spectrometer via a GC combustion III (Thermo Scientific), as described elsewhere (Brand, 1996; Sugimoto et al., 1991). Then, the apparent isotopic factor (α_app_) was calculated from δ^13^CH_4_ and δ^13^CO_2_ with the following equation:

α_app_ = (δ^13^CO_2_ + 10^3^) / (δ^13^CH_4_ + 10^3^)

If α_app_ is higher than 1.065, methane was mainly produced through the hydrogenotrophic pathway. On the other hand, if α_app_ is lower than 1.055, methane was produced through the acetoclastic pathway [2-3].

**Chemical analysis**

Acetic acid, butyric acid, and propionic acid were quantified in a ionic chromatograph (ICS 5000+, Thermo Fisher Scientific) equipped with a IonPAC ICE-AS1 column and using a mobile phase composed of heptafluorobutyric acid (0.4 mmol/L) and tetrabutylammonium (5 mmol/L).

Ammonium (NH_4_^+^) concentration was determined using the Nessler’s colorimetric method following the French standard (NF T 90-105) [4], and the dissolved organic and inorganic carbons (DOC and DIC, respectively) were measured following the French standard NF EN 1484 using a DOC analyser TOC-L-Shimadzu.

**16S rRNA sequencing**

Total RNA was extracted using the commercial kit FastRNA Pro™ Soil-Direct (MP Biomedicals) following the manufacturer’s specifications. Then, DNA co-extracted was removed using TURBO™ DNase (Ambion) kit following the manufacturer’s instructions. The RNA was denaturated by 2 min at 85°C in a dry bath and was then stored on ice. RNA purification was carried out using the RNAClean XP magnetic beads purification system (Beckman Coulter) by adding 1.8 volumes of beads by volume of RNA. After mixing by pipetting and 5 min of incubation, beads were captured using a magnetic rack on one side of the tube and then washed by adding 500 µL of 70% cold ethanol (diluted in DEPC-water). After incubation of the tube for 30 seconds at room temperature, the ethanol was removed. This washing step was repeated 3 times. Once ethanol evaporated, beads were resuspended with DEPC-water to elute RNA from the beads. Finally beads were removed using the magnetic rack and RNA was recovered in the supernatant. The integrity and quantity of the RNA was evaluated using the High Sensitivity RNA ScreenTape and 4200 TapeStation (Agilent Technologies) following the manufacturer’s protocol.

A reverse transcription PCR (RT-PCR) was carried out on the RNA using the mix iScript Reverse Transcription Supermix (Biorad) and the following thermocycler program: 5 min at 25°C, 30 min at 42°C and 5 min at 85°C. The cDNA was quantified using Qubit 2.0 fluorometer (ssDNA assay kit, Invitrogen, Life Technologies). The RT-PCR was used for the amplification of the bacterial and archaeal hypervariable region V4-V5 with the primers 515F (5′-GTGYCAGCMGCCGCGGTA-3′) and 928R (5′-CCCCGYCAATTCMTTTRAGT-3′) as previously described (Madigou et al., 2019). Briefly, a fusion method was employed (IonAmplicon Library Preparation (FusionMethod) Protocol, Revision C). The forward primer was modified by the addition of a PGM sequencing adaptor (adaptor A: 5′-CCATCTCATCCCTGCGTGTCTCCGACTCAG-3′) and a barcode (5′-adaptor ABarcode-515F-3′), and the reverse primer was modified by the addition of a PGM sequencing adaptor (adaptor trP1: 5′-CCTCTCTATGGGCAGTCGGTGAT-3′) (5′-adaptor trP1-928R-3′). The V4-V5 region was amplified according to Platinum Pfx protocol (Life Technologies). PCR products were cleaned using the Agencourt AMPure XP magnetic beads purification system (Beckman Coulter) and an emulsion PCR was carried out using the Ion PGM™ Template OT2 400 Kit with the Ion OneTouch™ 2 Instrument.

Sequencing was performed on an Ion Torrent Personal Genome Machine using an Ion 316 chip and the Ion PGM Sequencing 400 Kit following manufacturer’s instructions. The PGM software filtered out low quality and polyclonal sequence reads, and the quality-filtered data were exported as a FastQ file. 22,500–50,000 high-quality reads were generated for each sample. The sequencing data have been deposited in the bioproject PRJNA562430, and samples accession numbers go from SAMN12640739 to SAMN12640746, from SAMN12640748 to SAMN12640756, and from SAMN12640758 to SAMN12640759.

**Theory of Common Components Analysis (CCA) applied to 16S RNA sequencing data**

In CCA, a series of components descriptive of an active microbial population is obtained. Each component represents all the species showing a particular response to substrate, and is composed of a CCA scores vector, a CCA loadings vector, and a vector of saliences. CCA scores are representative of the substrate compositions associated with the microorganisms included in the active microbial populations reflected in the CCA loadings. The saliences indicate the weights of the corresponding variables in the construction of the CC.

**References**

1. Chapleur O, Bize A, Serain T, Mazéas L, Bouchez T. 2014. Co-inoculating ruminal content neither provides active hydrolytic microbes nor improves methanization of 13C-cellulose in batch digesters. FEMS Microbiol. Ecol. 87, 616–629. <https://doi.org/10.1111/1574-6941.12249>
2. Conrad R. 2005. Quantification of methanogenic pathways using stable carbon isotopic signatures: a review and a proposal. Org. Geochem. 36, 739–752. https://doi.org/10.1016/j.orggeochem.2004.09.006
3. Whiticar MJ, Faber E, Schoell M. 1986. Biogenic methane formation in marine and freshwater environments: CO2reduction vs. acetate fermentation-Isotope evidence. Geochim. Cosmochim. Acta 50, 693–709. <https://doi.org/10.1016/0016-7037(86)90346-7>
4. Cardona L, Levrard C, Guenne A, Chapleur O, Mazéas L. 2019. Co-digestion of wastewater sludge: Choosing the optimal blend. Waste Manag. 87, 772–781. https://doi.org/10.1016/J.WASMAN.2019.03.016
