# Supplementary material for: Assessment of the microbial interplay during anaerobic co-digestion of wastewater sludge using common components analysis
Source: PLoS One. 2020 May 1;15(5):e0232324. doi: 10.1371/journal.pone.0232324 (PMC7194399; doi:10.1371/journal.pone.0232324)
Supplement: S1 Table — (DOCX) [file pone.0232324.s006.docx]

PLOS ONE

**S1 Table.** Characteristics of substrates and inoculum

|  | **Wastewater Sludge** | **Fish**  **Waste** | **Garden**  **Grass** | **Inoculum** |
| --- | --- | --- | --- | --- |
| NH_4_^+^ (mgN/L) | 299 | 899 | 438 | 628 |
| DOC (mgC/L) | 1250 | 7921 | 7692 | 149 |
| DIC(mgC/L) | 99 | 346 | 424 | 753 |
| COD (gO2/L) | 103 | 310 | 95 | 13 |
| C (%) | 41.58 | 43.67 | 42.55 | 22.58 |
| N (%) | 2.46 | 9.50 | 2.20 | 2.19 |
| C/ N | 16.89 | 4.60 | 19.37 | 10.29 |
| Dry matter (DM) (%) | 5 | 24 | 11 | 1 |
| Volatile matter (VM) (%) | 81 | 79 | 84 | 61 |
| Lactate (mgC/L) | ND | ND | 398.80 | 23.40 |
| Formate (mgC/L) | ND | 138.52 | ND | ND |
| Acetate (mgC/L) | 537.08 | 62.88 | 11.08 | 2.08 |
| Propionate (mgC/L) | 441.83 | ND | ND | ND |
| Butyrate (mgC/L) | 199.20 | ND | ND | ND |
| Valerate (mgC/L) | 43.88 | ND | ND | ND |

ND : non-detected.
