# Supplementary material for: Assessment of the microbial interplay during anaerobic co-digestion of wastewater sludge using common components analysis
Source: PLoS One. 2020 May 1;15(5):e0232324. doi: 10.1371/journal.pone.0232324 (PMC7194399; doi:10.1371/journal.pone.0232324)
Supplement: S2 Table — (DOCX) [file pone.0232324.s007.docx]

PLOS ONE

S2 Table. Taxonomic affiliation for the selected OTUs in FW dataset.

| **OTU** | **Domain** | **Phylum** | **Class** | **Order** | **Family** | **Genus** | **species** | **Loading in CC1^a^** | **Loading in CC2^a^** |
| --- | --- | --- | --- | --- | --- | --- | --- | --- | --- |
| 63 | *Archaea* | *Euryarchaeota* | *Methanobacteria* | *Methanobacteriales* | *Methanobacteriaceae* | *Methanobacterium* | *unknown species* | + |  |
| 18 | *Archaea* | *Euryarchaeota* | *Methanomicrobia* | *Methanomicrobiales* | *Methanomicrobiaceae* | *Methanoculleus* | *unknown species* | + |  |
| 50 | *Archaea* | *Euryarchaeota* | *Methanomicrobia* | *Methanomicrobiales* | *Methanomicrobiaceae* | *Methanoculleus* | *unknown species* | + |  |
| 24 | *Archaea* | *Euryarchaeota* | *Methanomicrobia* | *Methanomicrobiales* | *Methanospirillaceae* | *Methanospirillum* | *unknown species* | + |  |
| 55 | *Archaea* | *Euryarchaeota* | *Methanomicrobia* | *Methanomicrobiales* | *Methanospirillaceae* | *Methanospirillum* | *Multi-affiliation* | + |  |
| 1 | *Archaea* | *Euryarchaeota* | *Methanomicrobia* | *Methanosarcinales* | *Methanosarcinaceae* | *Methanosarcina* | *Multi-affiliation* | + | - |
| 6 | *Archaea* | *Euryarchaeota* | *Methanomicrobia* | *Methanosarcinales* | *Methanosarcinaceae* | *Methanosarcina* | *unknown species* | - |  |
| 23 | *Bacteria* | *Bacteroidetes* | *Bacteroidia* | *Bacteroidales* | *Bacteroidaceae* | *Bacteroides* | *Multi-affiliation* | - | - |
| 88 | *Bacteria* | *Bacteroidetes* | *Bacteroidia* | *Bacteroidales* | *Bacteroidaceae* | *Bacteroides* | *Multi-affiliation* | - | - |
| 21 | *Bacteria* | *Bacteroidetes* | *Bacteroidia* | *Bacteroidales* | *Dysgonomonadaceae* | *Petrimonas* | *Multi-affiliation* | + | |
| 57 | *Bacteria* | *Bacteroidetes* | *Bacteroidia* | *Bacteroidales* | *Dysgonomonadaceae* | *Proteiniphilum* | *Multi-affiliation* | + |  |
| 152 | *Bacteria* | *Bacteroidetes* | *Bacteroidia* | *Bacteroidales* | *Dysgonomonadaceae* | *Proteiniphilum* | *unknown species* | + |  |
| 36 | *Bacteria* | *Bacteroidetes* | *Bacteroidia* | *Bacteroidales* | *GZKB124* | *unknown genus* | *unknown species* | + |  |
| 26 | *Bacteria* | *Bacteroidetes* | *Bacteroidia* | *Bacteroidales* | *Marinilabiliaceae* | *unknown genus* | *unknown species* | + | |
| 32 | *Bacteria* | *Bacteroidetes* | *Bacteroidia* | *Bacteroidales* | *Marinilabiliaceae* | *unknown genus* | *unknown species* | - | - |
| 112 | *Bacteria* | *Bacteroidetes* | *Bacteroidia* | *Bacteroidales* | *Paludibacteraceae* | *unknown genus* | *Multi-affiliation* | + | |
| 65 | *Bacteria* | *Bacteroidetes* | *Bacteroidia* | *Bacteroidales* | *Rikenellaceae* | *Blvii28 wastewater-sludge group* | *unknown species* | + | |
| 7 | *Bacteria* | *Bacteroidetes* | *Bacteroidia* | *Bacteroidales* | *Rikenellaceae* | *DMER64* | *unknown species* | + |  |
| 9 | *Bacteria* | *Bacteroidetes* | *Bacteroidia* | *Bacteroidales* | *Rikenellaceae* | *DMER64* | *Multi-affiliation* | + | - |
| 61 | *Bacteria* | *Bacteroidetes* | *Bacteroidia* | *Sphingobacteriales* | *Lentimicrobiaceae* | *unknown genus* | *Bacteroidetes bacterium ADurb.BinA012* | + | + |
| 40 | *Bacteria* | *Chlamydiae* | *LD1-PA32* | *unknown order* | *unknown family* | *unknown genus* | *unknown species* | + | + |
| 8 | *Bacteria* | *Chloroflexi* | *Anaerolineae* | *Anaerolineales* | *Anaerolineaceae* | *Flexilinea* | *unknown species* | + | + |
| 20 | *Bacteria* | *Chloroflexi* | *Anaerolineae* | *Anaerolineales* | *Anaerolineaceae* | *unknown genus* | *Multi-affiliation* | + |  |
| 4 | *Bacteria* | *Cloacimonetes* | *Cloacimonadia* | *Cloacimonadales* | *Cloacimonadaceae* | *Candidatus Cloacimonas* | *unknown species* | + | + |
| 27 | *Bacteria* | *Cloacimonetes* | *Cloacimonadia* | *Cloacimonadales* | *Cloacimonadaceae* | *Candidatus Cloacimonas* | *metagenome* | + |  |
| 12 | *Bacteria* | *Cloacimonetes* | *Cloacimonadia* | *Cloacimonadales* | *Cloacimonadaceae* | *W5* | *unknown species* | + | + |
| 13 | *Bacteria* | *Cloacimonetes* | *Cloacimonadia* | *Cloacimonadales* | *Cloacimonadaceae* | *W5* | *unknown species* | + | + |
| 30 | *Bacteria* | *Coprothermobacteraeota* | *Coprothermobacteria* | *Coprothermobacterales* | *Coprothermobacteraceae* | *Coprothermobacter* | *unknown species* | + |  |
| 3 | *Bacteria* | *Firmicutes* | *Clostridia* | *Clostridiales* | *Clostridiaceae 1* | *Clostridium sensu stricto 1* | *unknown species* | + | + |
| 2 | *Bacteria* | *Firmicutes* | *Clostridia* | *Clostridiales* | *Clostridiaceae 1* | *Clostridium sensu stricto 11* | *Multi-affiliation* | - | - |
| 29 | *Bacteria* | *Firmicutes* | *Clostridia* | *Clostridiales* | *Clostridiaceae 1* | *Clostridium sensu stricto 11* | *Multi-affiliation* | - | - |
| 5 | *Bacteria* | *Firmicutes* | *Clostridia* | *Clostridiales* | *Clostridiaceae 1* | *Clostridium sensu stricto 13* | *Multi-affiliation* | - | - |
| 128 | *Bacteria* | *Firmicutes* | *Clostridia* | *Clostridiales* | *Clostridiaceae 1* | *Clostridium sensu stricto 13* | *unknown species* | - |  |
| 114 | *Bacteria* | *Firmicutes* | *Clostridia* | *Clostridiales* | *Clostridiaceae 1* | *Clostridium sensu stricto 15* | *Multi-affiliation* | - | - |
| 22 | *Bacteria* | *Firmicutes* | *Clostridia* | *Clostridiales* | *Clostridiaceae 1* | *Hathewaya* | *bacterium NLAE-zl-G393* | - | + |
| 39 | *Bacteria* | *Firmicutes* | *Clostridia* | *Clostridiales* | *Clostridiaceae 1* | *Multi-affiliation* | *Multi-affiliation* | + |  |
| 86 | *Bacteria* | *Firmicutes* | *Clostridia* | *Clostridiales* | *Clostridiales vadinBB60 group* | *unknown genus* | *Multi-affiliation* | - |  |
| 51 | *Bacteria* | *Firmicutes* | *Clostridia* | *Clostridiales* | *Eubacteriaceae* | *Eubacterium* | *Eubacterium aggregans* | + | |
| 47 | *Bacteria* | *Firmicutes* | *Clostridia* | *Clostridiales* | *Family XI* | *Sporanaerobacter* | *Multi-affiliation* | - | - |
| 74 | *Bacteria* | *Firmicutes* | *Clostridia* | *Clostridiales* | *Family XI* | *unknown genus* | *unknown species* | - | - |
| 91 | *Bacteria* | *Firmicutes* | *Clostridia* | *Clostridiales* | *Family XI* | *unknown genus* | *unknown species* | - | - |
| 149 | *Bacteria* | *Firmicutes* | *Clostridia* | *Clostridiales* | *Family XI* | *unknown genus* | *unknown species* | - | - |
| 85 | *Bacteria* | *Firmicutes* | *Clostridia* | *Clostridiales* | *Lachnospiraceae* | *Cellulosilyticum* | *unknown species* | - | - |
| 49 | *Bacteria* | *Firmicutes* | *Clostridia* | *Clostridiales* | *Peptostreptococcaceae* | *Asaccharospora* | *Multi-affiliation* | - |  |
| 43 | *Bacteria* | *Firmicutes* | *Clostridia* | *Clostridiales* | *Peptostreptococcaceae* | *Intestinibacter* | *Multi-affiliation* | + |  |
| 14 | *Bacteria* | *Firmicutes* | *Clostridia* | *Clostridiales* | *Peptostreptococcaceae* | *Multi-affiliation* | *Multi-affiliation* | + |  |
| 68 | *Bacteria* | *Firmicutes* | *Clostridia* | *Clostridiales* | *Peptostreptococcaceae* | *Multi-affiliation* | *Multi-affiliation* | - |  |
| 73 | *Bacteria* | *Firmicutes* | *Clostridia* | *Clostridiales* | *Peptostreptococcaceae* | *Multi-affiliation* | *Multi-affiliation* | + |  |
| 102 | *Bacteria* | *Firmicutes* | *Clostridia* | *Clostridiales* | *Peptostreptococcaceae* | *Peptostreptococcus* | *Multi-affiliation* | + | |
| 53 | *Bacteria* | *Firmicutes* | *Clostridia* | *Clostridiales* | *Peptostreptococcaceae* | *Terrisporobacter* | *Multi-affiliation* | - | |
| 99 | *Bacteria* | *Firmicutes* | *Clostridia* | *Clostridiales* | *Ruminococcaceae* | *Pygmaiobacter* | *Pygmaiobacter massiliensis* | - | |
| 35 | *Bacteria* | *Firmicutes* | *Clostridia* | *Clostridiales* | *Ruminococcaceae* | *Ruminiclostridium* | *unknown species* | - | - |
| 15 | *Bacteria* | *Firmicutes* | *Clostridia* | *Clostridiales* | *Syntrophomonadaceae* | *Syntrophomonas* | *Multi-affiliation* | + | |
| 34 | *Bacteria* | *Firmicutes* | *Clostridia* | *Clostridiales* | *Syntrophomonadaceae* | *Syntrophomonas* | *unknown species* | - | + |
| 37 | *Bacteria* | *Firmicutes* | *Clostridia* | *Clostridiales* | *Syntrophomonadaceae* | *Syntrophomonas* | *Syntrophomonas wolfei* | + | - |
| 38 | *Bacteria* | *Firmicutes* | *Clostridia* | *Clostridiales* | *Syntrophomonadaceae* | *Syntrophomonas* | *unknown species* | + | |
| 45 | *Bacteria* | *Firmicutes* | *Clostridia* | *Clostridiales* | *Syntrophomonadaceae* | *Syntrophomonas* | *unknown species* | - | - |
| 56 | *Bacteria* | *Firmicutes* | *Clostridia* | *Clostridiales* | *Syntrophomonadaceae* | *Syntrophomonas* | *unknown species* | + |  |
| 33 | *Bacteria* | *Firmicutes* | *Clostridia* | *Clostridiales* | *Syntrophomonadaceae* | *unknown genus* | *anaerobic digester metagenome* | - | - |
| 115 | *Bacteria* | *Firmicutes* | *Clostridia* | *DTU014* | *unknown family* | *unknown genus* | *unknown species* | - |  |
| 31 | *Bacteria* | *Firmicutes* | *Erysipelotrichia* | *Erysipelotrichales* | *Erysipelotrichaceae* | *Turicibacter* | *Multi-affiliation* | + |  |
| 60 | *Bacteria* | *Hydrogenedentes* | *Hydrogenedentia* | *Hydrogenedentiales* | *Hydrogenedensaceae* | *unknown genus* | *unknown species* | + |  |
| 16 | *Bacteria* | *Planctomycetes* | *Phycisphaerae* | *MSBL9* | *SG8-4* | *unknown genus* | *unknown species* | + | - |
| 96 | *Bacteria* | *Planctomycetes* | *Phycisphaerae* | *MSBL9* | *SG8-4* | *unknown genus* | *unknown species* | + |  |
| 59 | *Bacteria* | *Proteobacteria* | *Deltaproteobacteria* | *Syntrophobacterales* | *Syntrophobacteraceae* | *Syntrophobacter* | *unknown species* | + |  |
| 28 | *Bacteria* | *Proteobacteria* | *Gammaproteobacteria* | *Betaproteobacteriales* | *Rhodocyclaceae* | *Azonexus* | *unknown species* | + |  |
| 41 | *Bacteria* | *Synergistetes* | *Synergistia* | *Synergistales* | *Synergistaceae* | *Aminobacterium* | *unknown species* | - |  |
| 11 | *Bacteria* | *Synergistetes* | *Synergistia* | *Synergistales* | *Synergistaceae* | *Multi-affiliation* | *Multi-affiliation* | + | + |
| 121 | *Bacteria* | *Synergistetes* | *Synergistia* | *Synergistales* | *Synergistaceae* | *Pyramidobacter* | *unknown species* | + | |
| 25 | *Bacteria* | *BRC1* | *unknown class* | *unknown order* | *unknown family* | *unknown genus* | *unknown species* | + | + |
| 205 | *Bacteria* | *WPS-2* | *unknown class* | *unknown order* | *unknown family* | *unknown genus* | *Burkholderiales bacterium Beta_02* | + | |

^a^Loading sign: ‘+’ refers to selected OTUs with positive loadings, and ‘-’ refers to selected OTUs with negative loadings. Loading signs are only provided for selected OTUs.
