# Supplementary material for: Assessment of the microbial interplay during anaerobic co-digestion of wastewater sludge using common components analysis
Source: PLoS One. 2020 May 1;15(5):e0232324. doi: 10.1371/journal.pone.0232324 (PMC7194399; doi:10.1371/journal.pone.0232324)
Supplement: S3 Table — (DOCX) [file pone.0232324.s008.docx]

PLOS ONE

S3 Table. Taxonomic affiliation for the selected OTUs in GG dataset.

| **OTU** | **Domain** | **Phylum** | **Class** | **Order** | **Family** | **Genus** | **species** | **Loading in CC1^a^** | **Loading in CC2^a^** |
| --- | --- | --- | --- | --- | --- | --- | --- | --- | --- |
| 93 | *Archaea* | *Euryarchaeota* | *Methanobacteria* | *Methanobacteriales* | *Methanobacteriaceae* | *Methanobacterium* | *Methanobacterium formicicum* | - |  |
| 63 | *Archaea* | *Euryarchaeota* | *Methanobacteria* | *Methanobacteriales* | *Methanobacteriaceae* | *Methanobacterium* | *unknown species* | + |  |
| 204 | *Archaea* | *Euryarchaeota* | *Methanobacteria* | *Methanobacteriales* | *Methanobacteriaceae* | *Methanobacterium* | *Multi-affiliation* | + |  |
| 1 | *Archaea* | *Euryarchaeota* | *Methanomicrobia* | *Methanosarcinales* | *Methanosarcinaceae* | *Methanosarcina* | *Multi-affiliation* | - | - |
| 64 | *Archaea* | *Euryarchaeota* | *Methanomicrobia* | *Methanomicrobiales* | *Methanomicrobiaceae* | *Methanofollis* | *unidentified archaeon* | - | - |
| 24 | *Archaea* | *Euryarchaeota* | *Methanomicrobia* | *Methanomicrobiales* | *Methanospirillaceae* | *Methanospirillum* | *unknown species* | - | + |
| 89 | *Archaea* | *Euryarchaeota* | *Methanomicrobia* | *Methanomicrobiales* | *Methanocorpusculaceae* | *Methanocorpusculum* | *Multi-affiliation* | - | + |
| 18 | *Archaea* | *Euryarchaeota* | *Methanomicrobia* | *Methanomicrobiales* | *Methanomicrobiaceae* | *Methanoculleus* | *unknown species* | + |  |
| 50 | *Archaea* | *Euryarchaeota* | *Methanomicrobia* | *Methanomicrobiales* | *Methanomicrobiaceae* | *Methanoculleus* | *unknown species* | + |  |
| 55 | *Archaea* | *Euryarchaeota* | *Methanomicrobia* | *Methanomicrobiales* | *Methanospirillaceae* | *Methanospirillum* | *Multi-affiliation* | | + |
| 136 | *Archaea* | *Euryarchaeota* | *Methanomicrobia* | *Methanomicrobiales* | *Methanocorpusculaceae* | *Methanocorpusculum* | *unknown species* | | + |
| 168 | *Bacteria* | *Acidobacteria* | *Aminicenantia* | *Aminicenantales* | *unknown family* | *unknown genus* | *unknown species* | | + |
| 78 | *Bacteria* | *Actinobacteria* | *Actinobacteria* | *Actinomycetales* | *Actinomycetaceae* | *Actinomyces* | *metagenome* |  | + |
| 132 | *Bacteria* | *Atribacteria* | *Caldatribacteriia* | *Caldatribacteriales* | *Caldatribacteriaceae* | *Candidatus Caldatribacterium* | *unknown species* | + |  |
| 21 | *Bacteria* | *Bacteroidetes* | *Bacteroidia* | *Bacteroidales* | *Dysgonomonadaceae* | *Petrimonas* | *Multi-affiliation* | - | - |
| 23 | *Bacteria* | *Bacteroidetes* | *Bacteroidia* | *Bacteroidales* | *Bacteroidaceae* | *Bacteroides* | *Multi-affiliation* | - | - |
| 76 | *Bacteria* | *Bacteroidetes* | *Bacteroidia* | *Bacteroidales* | *M2PB4-65 termite group* | *unknown genus* | *unknown species* | - | - |
| 125 | *Bacteria* | *Bacteroidetes* | *Bacteroidia* | *Bacteroidales* | *Paludibacteraceae* | *unknown genus* | *unknown species* | - | - |
| 69 | *Bacteria* | *Bacteroidetes* | *Bacteroidia* | *Bacteroidales* | *Rikenellaceae* | *dgA-11 gut group* | *unknown species* | - | + |
| 70 | *Bacteria* | *Bacteroidetes* | *Bacteroidia* | *Bacteroidales* | *Rikenellaceae* | *Rikenellaceae RC9 gut group* | *unknown species* | - | + |
| 75 | *Bacteria* | *Bacteroidetes* | *Bacteroidia* | *Bacteroidales* | *Dysgonomonadaceae* | *unknown genus* | *unknown species* | - | + |
| 101 | *Bacteria* | *Bacteroidetes* | *Bacteroidia* | *Bacteroidales* | *M2PB4-65 termite group* | *unknown genus* | *unknown species* | - | + |
| 66 | *Bacteria* | *Bacteroidetes* | *Bacteroidia* | *Bacteroidales* | *Dysgonomonadaceae* | *unknown genus* | *unknown species* | - |  |
| 67 | *Bacteria* | *Bacteroidetes* | *Bacteroidia* | *Bacteroidales* | *Rikenellaceae* | *DMER64* | *unknown species* | - |  |
| 84 | *Bacteria* | *Bacteroidetes* | *Bacteroidia* | *Bacteroidales* | *Tannerellaceae* | *Macellibacteroides* | *Multi-affiliation* | - |  |
| 118 | *Bacteria* | *Bacteroidetes* | *Bacteroidia* | *Bacteroidales* | *Bacteroidales UCG-001* | *unknown genus* | *unknown species* | - |  |
| 26 | *Bacteria* | *Bacteroidetes* | *Bacteroidia* | *Bacteroidales* | *Marinilabiliaceae* | *unknown genus* | *unknown species* | + | - |
| 7 | *Bacteria* | *Bacteroidetes* | *Bacteroidia* | *Bacteroidales* | *Rikenellaceae* | *DMER64* | *unknown species* | + | + |
| 9 | *Bacteria* | *Bacteroidetes* | *Bacteroidia* | *Bacteroidales* | *Rikenellaceae* | *DMER64* | *Multi-affiliation* | + | + |
| 36 | *Bacteria* | *Bacteroidetes* | *Bacteroidia* | *Bacteroidales* | *GZKB124* | *unknown genus* | *unknown species* | + | + |
| 57 | *Bacteria* | *Bacteroidetes* | *Bacteroidia* | *Bacteroidales* | *Dysgonomonadaceae* | *Proteiniphilum* | *Multi-affiliation* | + |  |
| 61 | *Bacteria* | *Bacteroidetes* | *Bacteroidia* | *Sphingobacteriales* | *Lentimicrobiaceae* | *unknown genus* | *Bacteroidetes bacterium ADurb.BinA012* | + |  |
| 112 | *Bacteria* | *Bacteroidetes* | *Bacteroidia* | *Bacteroidales* | *Paludibacteraceae* | *unknown genus* | *Multi-affiliation* | + |  |
| 140 | *Bacteria* | *Bacteroidetes* | *Bacteroidia* | *Bacteroidales* | *Rikenellaceae* | *Alistipes* | *Multi-affiliation* | + |  |
| 152 | *Bacteria* | *Bacteroidetes* | *Bacteroidia* | *Bacteroidales* | *Dysgonomonadaceae* | *Proteiniphilum* | *unknown species* | + |  |
| 32 | *Bacteria* | *Bacteroidetes* | *Bacteroidia* | *Bacteroidales* | *Marinilabiliaceae* | *unknown genus* | *unknown species* | | - |
| 248 | *Bacteria* | *Bacteroidetes* | *Bacteroidia* | *Bacteroidales* | *Marinilabiliaceae* | *Ruminofilibacter* | *Multi-affiliation* | | - |
| 380 | *Bacteria* | *Bacteroidetes* | *Bacteroidia* | *Bacteroidales* | *Bacteroidaceae* | *Bacteroides* | *Multi-affiliation* | | - |
| 430 | *Bacteria* | *Bacteroidetes* | *Bacteroidia* | *Bacteroidales* | *Rikenellaceae* | *Alistipes* | *Multi-affiliation* | | - |
| 543 | *Bacteria* | *Bacteroidetes* | *Bacteroidia* | *Bacteroidales* | *Rikenellaceae* | *Alistipes* | *Multi-affiliation* | | - |
| 48 | *Bacteria* | *Bacteroidetes* | *Bacteroidia* | *Sphingobacteriales* | *Lentimicrobiaceae* | *unknown genus* | *bacterium enrichment culture*  *clone R4-52B* | | + |
| 110 | *Bacteria* | *Bacteroidetes* | *Bacteroidia* | *Bacteroidales* | *Rikenellaceae* | *DMER64* | *Multi-affiliation* | | + |
| 133 | *Bacteria* | *Bacteroidetes* | *Bacteroidia* | *Bacteroidales* | *PeH15* | *unknown genus* | *unknown species* | | + |
| 158 | *Bacteria* | *Bacteroidetes* | *Bacteroidia* | *Bacteroidales* | *Tannerellaceae* | *Macellibacteroides* | *unknown species* | | + |
| 166 | *Bacteria* | *Bacteroidetes* | *Bacteroidia* | *Bacteroidales* | *Tannerellaceae* | *Parabacteroides* | *unknown species* | | + |
| 171 | *Bacteria* | *Bacteroidetes* | *Bacteroidia* | *Bacteroidales* | *Paludibacteraceae* | *unknown genus* | *unknown species* | | + |
| 40 | *Bacteria* | *Chlamydiae* | *LD1-PA32* | *unknown order* | *unknown family* | *unknown genus* | *unknown species* | + |  |
| 8 | *Bacteria* | *Chloroflexi* | *Anaerolineae* | *Anaerolineales* | *Anaerolineaceae* | *Flexilinea* | *unknown species* | + | - |
| 20 | *Bacteria* | *Chloroflexi* | *Anaerolineae* | *Anaerolineales* | *Anaerolineaceae* | *unknown genus* | *Multi-affiliation* | + | - |
| 159 | *Bacteria* | *Chloroflexi* | *Anaerolineae* | *RBG-13-54-9* | *unknown family* | *unknown genus* | *unknown species* | + |  |
| 211 | *Bacteria* | *Chloroflexi* | *Anaerolineae* | *Anaerolineales* | *Anaerolineaceae* | *unknown genus* | *unknown species* | + |  |
| 4 | *Bacteria* | *Cloacimonetes* | *Cloacimonadia* | *Cloacimonadales* | *Cloacimonadaceae* | *Candidatus Cloacimonas* | *unknown species* | - | + |
| 27 | *Bacteria* | *Cloacimonetes* | *Cloacimonadia* | *Cloacimonadales* | *Cloacimonadaceae* | *Candidatus Cloacimonas* | *metagenome* | - | + |
| 13 | *Bacteria* | *Cloacimonetes* | *Cloacimonadia* | *Cloacimonadales* | *Cloacimonadaceae* | *W5* | *unknown species* | + | - |
| 12 | *Bacteria* | *Cloacimonetes* | *Cloacimonadia* | *Cloacimonadales* | *Cloacimonadaceae* | *W5* | *unknown species* | + |  |
| 124 | *Bacteria* | *Cloacimonetes* | *Cloacimonadia* | *Cloacimonadales* | *Cloacimonadaceae* | *Candidatus Cloacimonas* | *metagenome* |  | + |
| 30 | *Bacteria* | *Coprothermobacteraeota* | *Coprothermobacteria* | *Coprothermobacterales* | *Coprothermobacteraceae* | *Coprothermobacter* | *unknown species* | + |  |
| 98 | *Bacteria* | *Cyanobacteria* | *Oxyphotobacteria* | *Chloroplast* | *unknown family* | *unknown genus* | *Multi-affiliation* | - |  |
| 62 | *Bacteria* | *Fibrobacteres* | *Fibrobacteria* | *Fibrobacterales* | *possible family 01* | *unknown genus* | *unknown species* | - | - |
| 58 | *Bacteria* | *Fibrobacteres* | *Fibrobacteria* | *Fibrobacterales* | *possible family 01* | *unknown genus* | *unknown species* | | + |
| 52 | *Bacteria* | *Firmicutes* | *Bacilli* | *Lactobacillales* | *Leuconostocaceae* | *Weissella* | *Multi-affiliation* | - | - |
| 87 | *Bacteria* | *Firmicutes* | *Bacilli* | *Lactobacillales* | *Enterococcaceae* | *Enterococcus* | *Multi-affiliation* | - | - |
| 71 | *Bacteria* | *Firmicutes* | *Bacilli* | *Lactobacillales* | *Leuconostocaceae* | *Leuconostoc* | *Multi-affiliation* | - |  |
| 138 | *Bacteria* | *Firmicutes* | *Bacilli* | *Lactobacillales* | *Leuconostocaceae* | *Weissella* | *Multi-affiliation* | - |  |
| 105 | *Bacteria* | *Firmicutes* | *Bacilli* | *Lactobacillales* | *Carnobacteriaceae* | *Trichococcus* | *Multi-affiliation* | | + |
| 5 | *Bacteria* | *Firmicutes* | *Clostridia* | *Clostridiales* | *Clostridiaceae 1* | *Clostridium sensu stricto 13* | *Multi-affiliation* | - | - |
| 53 | *Bacteria* | *Firmicutes* | *Clostridia* | *Clostridiales* | *Peptostreptococcaceae* | *Terrisporobacter* | *Multi-affiliation* | - | - |
| 77 | *Bacteria* | *Firmicutes* | *Clostridia* | *Clostridiales* | *Clostridiaceae 1* | *Clostridium sensu stricto 1* | *Multi-affiliation* | - | - |
| 80 | *Bacteria* | *Firmicutes* | *Clostridia* | *Clostridiales* | *Clostridiaceae 1* | *Clostridium sensu stricto 12* | *Multi-affiliation* | - | - |
| 109 | *Bacteria* | *Firmicutes* | *Clostridia* | *Clostridiales* | *Family XI* | *Sedimentibacter* | *unknown species* | - | - |
| 123 | *Bacteria* | *Firmicutes* | *Clostridia* | *Clostridiales* | *Lachnospiraceae* | *Butyrivibrio* | *unknown species* | - | - |
| 129 | *Bacteria* | *Firmicutes* | *Clostridia* | *Clostridiales* | *Clostridiaceae 1* | *Clostridium sensu stricto 1* | *Multi-affiliation* | - | - |
| 147 | *Bacteria* | *Firmicutes* | *Clostridia* | *Clostridiales* | *Lachnospiraceae* | *Lachnospira* | *unknown species* | - | - |
| 150 | *Bacteria* | *Firmicutes* | *Clostridia* | *Clostridiales* | *Christensenellaceae* | *Christensenellaceae R-7 group* | *Multi-affiliation* | - | - |
| 186 | *Bacteria* | *Firmicutes* | *Clostridia* | *Clostridiales* | *Clostridiaceae 1* | *Clostridium sensu stricto 13* | *Clostridium sp.* | - | - |
| 187 | *Bacteria* | *Firmicutes* | *Clostridia* | *Clostridiales* | *Christensenellaceae* | *Christensenellaceae R-7 group* | *Multi-affiliation* | - | - |
| 191 | *Bacteria* | *Firmicutes* | *Clostridia* | *Clostridiales* | *Lachnospiraceae* | *Herbinix* | *Multi-affiliation* | - | - |
| 207 | *Bacteria* | *Firmicutes* | *Clostridia* | *Clostridiales* | *Christensenellaceae* | *Christensenellaceae R-7 group* | *unknown species* | - | - |
| 227 | *Bacteria* | *Firmicutes* | *Clostridia* | *Clostridiales* | *Syntrophomonadaceae* | *Syntrophomonas* | *Multi-affiliation* | - | - |
| 250 | *Bacteria* | *Firmicutes* | *Clostridia* | *Clostridiales* | *Ruminococcaceae* | *[Eubacterium] coprostanoligenes group* | *unknown species* | - | - |
| 274 | *Bacteria* | *Firmicutes* | *Clostridia* | *Clostridiales* | *Ruminococcaceae* | *unknown genus* | *unknown species* | - | - |
| 10 | *Bacteria* | *Firmicutes* | *Clostridia* | *Clostridiales* | *Lachnospiraceae* | *unknown genus* | *unknown species* | - | + |
| 17 | *Bacteria* | *Firmicutes* | *Clostridia* | *Clostridiales* | *Syntrophomonadaceae* | *Syntrophomonas* | *Syntrophomonas curvata* | - | + |
| 51 | *Bacteria* | *Firmicutes* | *Clostridia* | *Clostridiales* | *Eubacteriaceae* | *Eubacterium* | *Eubacterium aggregans* | - | + |
| 54 | *Bacteria* | *Firmicutes* | *Clostridia* | *Clostridiales* | *Christensenellaceae* | *Christensenellaceae R-7 group* | *unknown species* | - | + |
| 106 | *Bacteria* | *Firmicutes* | *Clostridia* | *Clostridiales* | *Christensenellaceae* | *Christensenellaceae R-7 group* | *unknown species* | - | + |
| 34 | *Bacteria* | *Firmicutes* | *Clostridia* | *Clostridiales* | *Syntrophomonadaceae* | *Syntrophomonas* | *unknown species* | - |  |
| 42 | *Bacteria* | *Firmicutes* | *Clostridia* | *Clostridiales* | *Peptococcaceae* | *Pelotomaculum* | *unknown species* | - |  |
| 107 | *Bacteria* | *Firmicutes* | *Clostridia* | *Clostridiales* | *Lachnospiraceae* | *Multi-affiliation* | *Multi-affiliation* | - |  |
| 120 | *Bacteria* | *Firmicutes* | *Clostridia* | *Clostridiales* | *Eubacteriaceae* | *Eubacterium* |  | - |  |
| 155 | *Bacteria* | *Firmicutes* | *Clostridia* | *Clostridiales* | *Christensenellaceae* | *Christensenellaceae R-7 group* | *unknown species* | - |  |
| 165 | *Bacteria* | *Firmicutes* | *Clostridia* | *Clostridiales* | *Clostridiales vadinBB60 group* | *unknown genus* | *Multi-affiliation* | - |  |
| 233 | *Bacteria* | *Firmicutes* | *Clostridia* | *Clostridiales* | *Ruminococcaceae* | *Ruminiclostridium 1* | *unknown species* | - |  |
| 37 | *Bacteria* | *Firmicutes* | *Clostridia* | *Clostridiales* | *Syntrophomonadaceae* | *Syntrophomonas* | *Syntrophomonas wolfei* | + | - |
| 3 | *Bacteria* | *Firmicutes* | *Clostridia* | *Clostridiales* | *Clostridiaceae 1* | *Clostridium sensu stricto 1* | *unknown species* | + |  |
| 14 | *Bacteria* | *Firmicutes* | *Clostridia* | *Clostridiales* | *Peptostreptococcaceae* | *Multi-affiliation* | *Multi-affiliation* | + |  |
| 15 | *Bacteria* | *Firmicutes* | *Clostridia* | *Clostridiales* | *Syntrophomonadaceae* | *Syntrophomonas* | *Multi-affiliation* | + |  |
| 38 | *Bacteria* | *Firmicutes* | *Clostridia* | *Clostridiales* | *Syntrophomonadaceae* | *Syntrophomonas* | *unknown species* | + |  |
| 39 | *Bacteria* | *Firmicutes* | *Clostridia* | *Clostridiales* | *Clostridiaceae 1* | *Multi-affiliation* | *Multi-affiliation* | + |  |
| 43 | *Bacteria* | *Firmicutes* | *Clostridia* | *Clostridiales* | *Peptostreptococcaceae* | *Intestinibacter* | *Multi-affiliation* | + |  |
| 56 | *Bacteria* | *Firmicutes* | *Clostridia* | *Clostridiales* | *Syntrophomonadaceae* | *Syntrophomonas* | *unknown species* | + |  |
| 73 | *Bacteria* | *Firmicutes* | *Clostridia* | *Clostridiales* | *Peptostreptococcaceae* | *Multi-affiliation* | *Multi-affiliation* | + |  |
| 117 | *Bacteria* | *Firmicutes* | *Clostridia* | *Clostridiales* | *Syntrophomonadaceae* | *Syntrophomonas* | *unknown species* | + |  |
| 202 | *Bacteria* | *Firmicutes* | *Clostridia* | *Clostridiales* | *Lachnospiraceae* | *Anaerostipes* | *unknown species* | + |  |
| 68 | *Bacteria* | *Firmicutes* | *Clostridia* | *Clostridiales* | *Peptostreptococcaceae* | *Multi-affiliation* | *Multi-affiliation* | | - |
| 99 | *Bacteria* | *Firmicutes* | *Clostridia* | *Clostridiales* | *Ruminococcaceae* | *Pygmaiobacter* | *Pygmaiobacter massiliensis* | | - |
| 100 | *Bacteria* | *Firmicutes* | *Clostridia* | *Clostridiales* | *Clostridiaceae 1* | *Clostridium sensu stricto 1* | *Multi-affiliation* | | - |
| 128 | *Bacteria* | *Firmicutes* | *Clostridia* | *Clostridiales* | *Clostridiaceae 1* | *Clostridium sensu stricto 13* | *unknown species* | | - |
| 183 | *Bacteria* | *Firmicutes* | *Clostridia* | *Clostridiales* | *Clostridiaceae 1* | *Clostridium sensu stricto 1* | *Multi-affiliation* | | - |
| 285 | *Bacteria* | *Firmicutes* | *Clostridia* | *Clostridiales* | *Christensenellaceae* | *Christensenellaceae R-7 group* | *unknown species* | | - |
| 315 | *Bacteria* | *Firmicutes* | *Clostridia* | *Clostridiales* | *Ruminococcaceae* | *Butyricicoccus* | *Multi-affiliation* | | - |
| 324 | *Bacteria* | *Firmicutes* | *Clostridia* | *Clostridiales* | *Ruminococcaceae* | *Ruminococcaceae UCG-013* | *unknown species* | | - |
| 384 | *Bacteria* | *Firmicutes* | *Clostridia* | *Clostridiales* | *Gracilibacteraceae* | *Lutispora* | *Clostridium* |  | - |
| 557 | *Bacteria* | *Firmicutes* | *Clostridia* | *Clostridiales* | *Clostridiales vadinBB60 group* | *unknown genus* | *unknown species* | | - |
| 604 | *Bacteria* | *Firmicutes* | *Clostridia* | *Clostridiales* | *Clostridiaceae 1* | *Clostridium sensu stricto 3* | *Multi-affiliation* | | - |
| 81 | *Bacteria* | *Firmicutes* | *Clostridia* | *Clostridiales* | *Eubacteriaceae* | *Acetobacterium* | *Multi-affiliation* | | + |
| 102 | *Bacteria* | *Firmicutes* | *Clostridia* | *Clostridiales* | *Peptostreptococcaceae* | *Peptostreptococcus* | *Multi-affiliation* | | + |
| 151 | *Bacteria* | *Firmicutes* | *Clostridia* | *Clostridiales* | *Christensenellaceae* | *Christensenellaceae R-7 group* | *unknown species* | | + |
| 189 | *Bacteria* | *Firmicutes* | *Clostridia* | *Clostridiales* | *Christensenellaceae* | *Christensenellaceae R-7 group* | *unknown species* | | + |
| 210 | *Bacteria* | *Firmicutes* | *Clostridia* | *Clostridiales* | *Ruminococcaceae* | *Ruminococcaceae UCG-008* | *unknown species* | | + |
| 214 | *Bacteria* | *Firmicutes* | *Clostridia* | *Clostridiales* | *Ruminococcaceae* | *Ruminococcaceae NK4A214 group* | *unknown species* | | + |
| 218 | *Bacteria* | *Firmicutes* | *Clostridia* | *Clostridiales* | *Christensenellaceae* | *Christensenellaceae R-7 group* | *unknown species* | | + |
| 238 | *Bacteria* | *Firmicutes* | *Clostridia* | *Clostridiales* | *Christensenellaceae* | *Christensenellaceae R-7 group* | *unknown species* | | + |
| 240 | *Bacteria* | *Firmicutes* | *Clostridia* | *Clostridiales* | *Christensenellaceae* | *Christensenellaceae R-7 group* | *unknown species* | | + |
| 246 | *Bacteria* | *Firmicutes* | *Clostridia* | *Clostridiales* | *Ruminococcaceae* | *unknown genus* | *unknown species* | | + |
| 263 | *Bacteria* | *Firmicutes* | *Clostridia* | *Clostridiales* | *Christensenellaceae* | *Christensenellaceae R-7 group* | *Multi-affiliation* | | + |
| 322 | *Bacteria* | *Firmicutes* | *Clostridia* | *Clostridiales* | *Christensenellaceae* | *Christensenellaceae R-7 group* | *unknown species* | | + |
| 31 | *Bacteria* | *Firmicutes* | *Erysipelotrichia* | *Erysipelotrichales* | *Erysipelotrichaceae* | *Turicibacter* | *Multi-affiliation* | + |  |
| 60 | *Bacteria* | *Hydrogenedentes* | *Hydrogenedentia* | *Hydrogenedentiales* | *Hydrogenedensaceae* | *unknown genus* | *unknown species* | + |  |
| 170 | *Bacteria* | *Lentisphaerae* | *Lentisphaeria* | *Victivallales* | *Victivallaceae* | *unknown genus* | *unknown species* | - | - |
| 119 | *Bacteria* | *Lentisphaerae* | *Oligosphaeria* | *Oligosphaerales* | *Oligosphaeraceae* | *Oligosphaera* | *Multi-affiliation* | + |  |
| 220 | *Bacteria* | *Lentisphaerae* | *Oligosphaeria* | *Oligosphaerales* | *Oligosphaeraceae* | *Z20* | *unknown species* | | + |
| 16 | *Bacteria* | *Planctomycetes* | *Phycisphaerae* | *MSBL9* | *SG8-4* | *unknown genus* | *unknown species* | + | + |
| 96 | *Bacteria* | *Planctomycetes* | *Phycisphaerae* | *MSBL9* | *SG8-4* | *unknown genus* | *unknown species* | + |  |
| 154 | *Bacteria* | *Planctomycetes* | *Phycisphaerae* | *Pla1 lineage* | *unknown family* | *unknown genus* | *unknown species* | + |  |
| 162 | *Bacteria* | *Planctomycetes* | *Phycisphaerae* | *mle1-8* | *unknown family* | *unknown genus* | *unknown species* | + |  |
| 139 | *Bacteria* | *Planctomycetes* | *Phycisphaerae* | *MSBL9* | *SM23-30* | *unknown genus* | *unknown species* | | + |
| 103 | *Bacteria* | *Planctomycetes* | *Planctomycetacia* | *Pirellulales* | *Pirellulaceae* | *Pir4 lineage* | *unknown species* | + |  |
| 157 | *Bacteria* | *Proteobacteria* | *Alphaproteobacteria* | *Rhodobacterales* | *Rhodobacteraceae* | *Multi-affiliation* | *Multi-affiliation* | | + |
| 164 | *Bacteria* | *Proteobacteria* | *Alphaproteobacteria* | *Rhodospirillales* | *unknown family* | *unknown genus* | *unknown species* | | + |
| 59 | *Bacteria* | *Proteobacteria* | *Deltaproteobacteria* | *Syntrophobacterales* | *Syntrophobacteraceae* | *Syntrophobacter* | *unknown species* | + |  |
| 83 | *Bacteria* | *Proteobacteria* | *Deltaproteobacteria* | *Oligoflexales* | *053A03-B-DI-P58* | *unknown genus* | *unknown species* | | + |
| 104 | *Bacteria* | *Proteobacteria* | *Gammaproteobacteria* | *Enterobacteriales* | *Enterobacteriaceae* | *Multi-affiliation* | *Multi-affiliation* | - | - |
| 194 | *Bacteria* | *Proteobacteria* | *Gammaproteobacteria* | *Enterobacteriales* | *Enterobacteriaceae* | *Escherichia-Shigella* | *Multi-affiliation* | - | - |
| 82 | *Bacteria* | *Proteobacteria* | *Gammaproteobacteria* | *Betaproteobacteriales* | *Rhodocyclaceae* | *Dechlorobacter* | *unknown species* | + |  |
| 408 | *Bacteria* | *Proteobacteria* | *Gammaproteobacteria* | *Enterobacteriales* | *Enterobacteriaceae* | *Multi-affiliation* | *Multi-affiliation* | | - |
| 28 | *Bacteria* | *Proteobacteria* | *Gammaproteobacteria* | *Betaproteobacteriales* | *Rhodocyclaceae* | *Azonexus* | *unknown species* | | + |
| 46 | *Bacteria* | *Proteobacteria* | *Gammaproteobacteria* | *Betaproteobacteriales* | *Rhodocyclaceae* | *Thauera* | *Multi-affiliation* | | + |
| 79 | *Bacteria* | *Spirochaetes* | *Spirochaetia* | *Spirochaetales* | *Spirochaetaceae* | *Treponema 2* | *Multi-affiliation* | - | - |
| 92 | *Bacteria* | *Spirochaetes* | *Spirochaetia* | *Spirochaetales* | *Spirochaetaceae* | *Treponema 2* | *metagenome* | - |  |
| 144 | *Bacteria* | *Spirochaetes* | *Spirochaetia* | *Spirochaetales* | *Spirochaetaceae* | *Sphaerochaeta* | *unknown species* | | + |
| 11 | *Bacteria* | *Synergistetes* | *Synergistia* | *Synergistales* | *Synergistaceae* | *Multi-affiliation* | *Multi-affiliation* | + |  |
| 97 | *Bacteria* | *Synergistetes* | *Synergistia* | *Synergistales* | *Synergistaceae* | *Syner-01* | *Multi-affiliation* | + |  |
| 121 | *Bacteria* | *Synergistetes* | *Synergistia* | *Synergistales* | *Synergistaceae* | *Pyramidobacter* | *unknown species* | | + |
| 177 | *Bacteria* | *Tenericutes* | *Mollicutes* | *Izimaplasmatales* | *unknown family* | *unknown genus* | *unknown species* | - | - |
| 245 | *Bacteria* | *Tenericutes* | *Mollicutes* | *Izimaplasmatales* | *unknown family* | *unknown genus* | *unknown species* | | - |
| 90 | *Bacteria* | *Thermotogae* | *Thermotogae* | *Petrotogales* | *Petrotogaceae* | *AUTHM297* | *metagenome* | - |  |
| 44 | *Bacteria* | *Thermotogae* | *Thermotogae* | *Petrotogales* | *Petrotogaceae* | *Defluviitoga* | *Multi-affiliation* | + |  |
| 25 | *Bacteria* | *BRC1* | *unknown class* | *unknown order* | *unknown family* | *unknown genus* | *unknown species* | + |  |
| 127 | *Bacteria* | *Armatimonadetes* | *unknown class* | *unknown order* | *unknown family* | *unknown genus* | *unknown species* | + |  |
| 135 | *Bacteria* | *BRC1* | *unknown class* | *unknown order* | *unknown family* | *unknown genus* | *unknown species* | + |  |

^a^Loading sign: ‘+’ refers to selected OTUs with positive loadings, and ‘-’ refers to selected OTUs with negative loadings. Loading signs are only provided for selected OTUs.
