# Supplementary material for: Assessment of the microbial interplay during anaerobic co-digestion of wastewater sludge using common components analysis
Source: PLoS One. 2020 May 1;15(5):e0232324. doi: 10.1371/journal.pone.0232324 (PMC7194399; doi:10.1371/journal.pone.0232324)
Supplement: S1 Data — (PDF) [file pone.0232324.s009.pdf]

Waste Management 87 (2019) 772–781

Contents lists available at ScienceDirect

Waste Management

journal homepage: www.elsevier.com/locate/wasman

Co-digestion of wastewater sludge: Choosing the optimal blend

Laëtitia Cardona, Camille Levrard, Angeline Guenne, Olivier Chapleur *, Laurent Mazéas

Hydrosystems and Bioprocesses Research Unit, Irstea, 1 rue Pierre-Gilles de Gennes, CS 10030, 92761 Antony Cedex, France

ARTICLE INFO

Article history:
Received 22 August 2018
Revised 6 March 2019
Accepted 7 March 2019
Available online 15 March 2019

Keywords:
Anaerobic digestion
Methanogenic pathways
Grass
Fish
Carbon-isotopic fractionation

ABSTRACT

Anaerobic co-digestion (AcoD) is a promising strategy to increase the methane production of anaerobic digestion plants treating wastewater sludge (WAS). In this work the degradability of six different mixtures of WAS with fish waste (FW) or garden-grass (GG) was evaluated and compared to the three mono-digestions. Degradation performances and methanogenic pathways, determined with the isotopic signatures of biogas, were compared across time. Fish and grass mono-digestion provided a higher final methane production than WAS mono-digestion. In co-digestion the addition of 25% of fish was enough to increase the final methane production from WAS while 50% of grass was necessary. To determine the optimal blend of WAS co-digestion two indicators were specifically designed, representing the maximum potential production (ODI) and the expected production in mono-digestion conditions (MDI). The comparison between these indicators and the experimental results showed that the most productive blend was composed of 75% of co-substrate, fish or grass, with WAS. Indeed, the final methane production was increased by 1.9 times with fish and by 1.7 times with grass associated to an increase of the methane production rate by 1.5 times. Even if the same succession of methanogenic pathways across time was observed for the different mixtures, their relative proportions were different. Sewage sludge degradation was mostly achieved through hydrogenotrophic pathway while acetoclastic pathway was dominant for fish and grass degradation. These results were confirmed by the identification of Archaea with 16S sequencing.

© 2019 Elsevier Ltd. All rights reserved.

1. Introduction

Anaerobic Digestion (AD) is a multistep biological process allowing to convert various types of organic waste into a renewable energy, the biogas (composed of CH_4 and CO_2) and digestate. This bioprocess has been used for more than a century, in particular to stabilise the wastewater sludge (WAS) obtained from wastewater treatment plants (Astals et al., 2012). It is particularly attractive as it allows to simultaneously produce energy in the form of biogas and to reduce the volume of sludge (Luostarinen et al., 2009). However, the benefit of using only WAS to produce methane by AD is limited by its low C/N ratio and low digestion efficiency, leading to low CH_4 production yield (Astals et al., 2013; Park et al., 2016).

A way to overcome this major drawback is to balance the low C/N ratio by mixing wastewater sludge with other substrates richer in carbon. This strategy of mixing different types of substrates, or performing anaerobic co-digestion (AcoD), has multiple advantages as: (1) improving the performances of digesters treating wastewater sludge (Mata-Alvarez et al., 2014), (2) treating several types of waste at the same time and 3) limiting the risk of inhibition that can occur during mono-digestion with the production of inhibitors for example (Borowski and Kubacki, 2015). Almost any type of organic waste can be treated by AD but each of them has specific properties which can bring some advantages and disadvantages. For example, animal manure and slaughterhouse waste have a high organic content but usually cause process disturbances in mono-digestion because of their high proteins and/or lipids content. This can lead to the accumulation of ammonia, volatile fatty acids (VFA) and long chain fatty acids (LCFA) (Hansen et al., 1998; Pitk et al., 2013, Borowski and Kubacki, 2015) known to be responsible of process failure. WAS as a co-substrate for these waste allows to dilute compounds potentially leading to inhibition, such as proteins and lipids and limit the risk of inhibition by a too fast acidification thanks to the high buffer capacity of the WAS (Prabhu and Mutnuri, 2016).

Different waste have already been successfully tested to improve WAS anaerobic digestion. In association with slaughterhouse waste, Borowski and Kubacki (2015) succeeded in increasing the specific methane production by 2-fold when 50% of slaughterhouse waste

* Corresponding author.
E-mail addresses: laetitia.cardona@irstea.fr (L. Cardona), camille.levrard@irstea.fr (C. Levrard), angeline.guenne@irstea.fr (A. Guenne), olivier.chapleur@irstea.fr (O. Chapleur), laurent.mazeas@irstea.fr (L. Mazéas).

https://doi.org/10.1016/j.wasman.2019.03.016
0956-053X/© 2019 Elsevier Ltd. All rights reserved.

was mixed to WAS at an organic loading rate (OLR) of 4 kgVS/m^{3}d. Neither ammonia nor LCFA inhibition was observed and an effect of VFA accumulation was observed only when the OLR was superior to 4 kgVS/m^{3}d. Wickham et al. (2016) tested several waste such as food waste, paper pulp, fat-grease-oil (FOG) waste and dehydrated *Ulva* macroalgae. Each substrate was mixed at different ratio with WAS (5, 10 and 15% by weight). Final methane production was increased by three to six times thanks to the co-digestion compared to mono-digestion of WAS.

In this study, different mixtures of wastewater sludge (WAS) with garden-grass (GG) or fish waste (FW) as co-substrates were tested to determine the optimal blend allowing for the most efficient CH_4 production. Total fish production in the world has expanded since the last five decades from 20 million tons in 1960 to 167.2 million tons in 2014 mainly due to the increase of the aquaculture production (FAO, 2016). The amount of waste provided by the fishery industries (as canneries) is important. Some authors evaluated the possibility to use different parts of the fish (skins, viscera, bones ...) (Donoso-Bravo et al., 2015), or different fish species (Eiroa et al., 2012) for methane production with high biodegradability level. However, this type of waste can be rich in protein and/or lipids inducing ammonia and/or LCFA inhibition as observed by Eiroa et al. (2012). Literature on the possibility to use fish waste as co-substrate in AD is still scarce and to the best of our knowledge co-digestion of fish waste with wastewater sludge was not studied yet.

Grass has a high potential as renewable biomass source due to its high biodegradability and biogas production potential (Dai et al., 2016; Prochnow et al., 2009). Using grass as a feedstock can lead to an ammonia accumulation due to the high protein content of certain types of green waste (Prochnow et al., 2009; Ward et al., 2008). Several investigations were conducted on the anaerobic co-digestion (AcoD) of WAS and grass and showed an enhancement of methane content (Dai et al., 2016; Hidaka et al., 2013). Nonetheless further studies are needed to understand the effect of the grass addition in order to optimise the methane production.

The aim of this study is to investigate the possibility for improving wastewater sludge degradation during anaerobic co-digestion with fish waste or garden grass. Degradation performances and methanogenic pathway, determined with the isotopic signatures of biogas (Conrad, 2005), were compared across time. Two biodegration indicators were specifically desgined to determine the mixes enabled to improve the methane production. As far we know the impact of the anaerobic co-digestion on the methanogenic pathway monitored by the isotopic analysis has not been studied yet.

2. Methods

2.1. Feedstock preparation and characterisation

Wastewater sludge came from an industrial wastewater treatment plant (Valenton, France). Two organic co-substrates were tested. Fish waste was collected from a fish shop and grass from the mowing of the Institute's lawn. Both waste were crushed and the solid part was stored at 4 °C during two days before they were used.

The inoculum came from a mesophilic full scale anaerobic digester treating primary sludge at the Valenton (France) wastewater treatment plant. In order to degrade the residual organic matter in excess it was stored at 35 °C during two weeks in anaerobic condition without feeding before being used.

All substrates and inoculum were characterised by different chemical analyses and the results are summarised in the Table 1.

2.2. Co-digestion experimental set-up

In total 27 anaerobic batch bioreactors were set-up using 1 L glass bottles (700 mL working volume). Each digester was inoculated with methanogenic sludge and fed with a mixture of a main substrate (wastewater sludge) and one co-substrate (fish waste or grass) to reach a substrate/inoculum ratio of 12 gCOD/1.2 gCOD. Different ratios of main substrate/co-substrate were tested (25/75, 50/50, 75/25) as detailed in the Supplementary Table A.1. Controls with 100% of wastewater sludge, fish waste or grass were also carried out. All the digesters were complemented with a biochemical potential buffer (International Standard ISO 11,734 (1995)) to reach a final working volume of 700 mL. All incubations were performed in triplicate. The bioreactors were then sealed with a screw cap and a rubber septum. The headspaces were flushed with N_2 (purity > 99.99%, Linde gas SA) and the bottles were incubated at 35 °C in the dark and without agitation.

Weekly, 6 mL of liquid phase were sampled through the septum using a syringe and centrifuged at 10,000 g for 10 min. The supernatant and he pellet were snap frozen and kept at −20 °C for chemical analysis and −80 °C for microbial analysis.

2.3. Biochemical methane potential (BMP) experimental setup

BMP tests were carried out for each substrate in mono-digestion to assess their methane maximal production under optimal parameters. The ratio substrate/inoculum used in BMP test was 0.7 gCOD/7 gCOD to limit the latency due to the microbial

Table 1
Characteristics of substrates and inoculum.

	Wastewater sludge	Fish	Grass	Inoculum
NH_4^+ (mgN/L)	299	899	438	628
DOC (mgC/L)	1250	7921	7692	149
DIC (mgC/L)	99	346	424	753
COD (gO2/L)	103	310	95	13
C (%)	41.58	43.67	42.55	22.58
N (%)	2.46	9.50	2.20	2.19
C/N	16.89	4.60	19.37	10.29
Dry matter (DM) (%)	5	24	11	1
Volatile matter (VM) (%)	81	79	84	61
Lactate (mgC/L)	0.00	0.00	398.80	23.40
Formate (mgC/L)	0.00	138.52	0.00	0.00
Acetate (mgC/L)	537.08	62.88	11.08	2.08
Propionate (mgC/L)	441.83	0.00	0.00	0.00
Butyrate (mgC/L)	199.20	0.00	0.00	0.00
Valerate (mgC/L)	43.88	0.00	0.00	0.00

growth. The biochemical potential buffer previously mentioned was used to reach a final volume of 500 mL in 1L glass bottles. As for the batch experiment, bottles were sealed, flushed with N_2 and incubated at 35 °C in the dark without agitation. The experiment was made in triplicate. Gas production and composition were followed over time. A control containing only the inoculum was carried out in parallel and the biogas production of this control was taken into account to calculate the substrates gas productions. The mixtures details are presented in the Supplementary Table A.1.

2.4. Gas production and stable carbon isotope signature

The biogas accumulation in the headspace was measured using a differential manometer (Digitron 2082P). The biogas was then analysed directly in the headspace using a micro gas chromatograph (CP4900, Varian) as described in Chapleur et al. (2014). Data were used to calculate the biogas production at standard temperature and pressure. Different parameters used to quantify the methane production potential were calculated using R CRAN software and the Gompertz equation with Grofit package as described in Poirier et al. (2016):

$$y(t) = A.\exp[-\exp\left(\frac{\mu.e}{A}(\lambda - t) + 1\right)]$$

where y (t) is a cumulative CH_4 production (mL), A is the ultimate CH_4 yield (mL), μ is the maximum production of CH_4 production rate (mL/day), and λ is the lag phase (day).

The methanogenic pathways during the substrates degradation (acetoclastic methanogenesis or hydrogenotrophic methanogenesis) were determined by the gas isotopic signature analysis. Periodically gas was sampled into a 7 mL vacuumed serum tubes for analysis of $\delta^{13}CH_4$ and $\delta^{13}CO_2$. A Trace Gas Chromatograph Ultra (Thermo Scientific) attached to a Delta V Plus isotope ratio mass spectrometer via a GC combustion III (Thermo Scientific) was used to carry out the analysis. The principle of the method was described by (Brand, 1996; Sugimoto et al., 1991). The uncertainties, determined by replicate measurement, for $\delta^{13}CH_4$ and $\delta^{13}CO_2$ analysis was around 0.5‰. As indicator of the methanogenic pathway, the apparent isotopic factor (α_{app}) was calculated as presented in the following equation:

$$\alpha_{app} = (\delta^{13}CO_2 + 10^3)/(\delta^{13}CH_4 + 10^3)$$

It is usually assumed that if the α_{app} is superior to 1.065, the hydrogenotrophic way is the most important. On the contrary if the α_{app} is inferior to 1.055, the methanogenesis is dominated by the acetoclastic way (Conrad, 2005; Whiticar et al., 1986).

2.5. Chemical analysis

Volatile Fatty Acids (VFA) concentrations were measured using ionic chromatography (ICS 5000+, Thermo Fisher Scientific) equipped with IonPAC ICE-AS1 column. The mobile phase was composed of heptafluorobutyric acid (0.4 mmol/L) and tetrabutylammonium (5 mmol/L). The VFA quantified were acetate, propionate, butyrate, valerate, formate, lactate and caproate.

Ammonium (NH_4^+) concentration was measured using the Nessler's colorimetric method following the French standard (NF T 90-105) in spectroscopic tanks using Hach spectrometer DR2800. The link between Free Ammonia Nitrogen (FAN), Total Ammonia Nitrogen (TAN), pH and temperature can be summarized with the following equation (Anthonisen et al., 1976):

$$FAN = \frac{10^{pH}}{(\exp\left(\frac{6344}{T}\right) + 10^{pH})} xTAN$$

where T is the temperature in Kelvin.

Dissolved organic and inorganic (DOC and DIC) carbons were measured following the French standard NF EN 1484 using a DOC analyser TOC-L Shimadzu.

Chemical oxygen demand (COD) was measured with LCK514 kit (Hach Lange) according to the manufacturer's instructions.

The carbon and nitrogen quantities contained in the substrates and inoculum were analysed on the crushed and dried sample. 10 mg of the sample was placed on sampler tin and analysed using an elementary analyser (VARIO EL III, Bioritech).

2.6. RNA extraction and 16S RNA sequencing analysis

Based on the methane production (Fig. 2), a total of 19 samples were selected. Total RNA was extracted using the commercial kit FastRNA Pro™ Soil-Direct (MP Biomedicals) following the manufacturer's specifications. Then, DNA co-extracted was removed using TURBO™ DNase (Ambion) kit following the manufacturer's instructions. The RNA was denaturated by 2 min at 85 °C in a dry bath and was then stored on ice. RNA purification was carried out using the Agencourt AMpure RNA magnetic beads purification system (Beckman Coulter) by adding 1.8 volumes of beads by volume of RNA. After mixing by pipetting and 5 min of incubation, beads were captured using a magnetic rack on one side of the tube and then washed by adding 500 μL of 70% cold ethanol (diluted in DEPC-water). After incubation of the tube during 30 s at room temperature, the ethanol was removed. This washing step was repeated 3 times. Once ethanol finally evaporated, beads were resuspended with DEPC-water to eluted RNA from the beads. Finally beads were removed using the magnetic rack and RNA was recovered in the supernatant. The integrity and quantity of the RNA was evaluated using the Hight Sensitivity RNA ScreenTape and 4200 TapeStation (Agilent Technologies) following the manufacturer's protocol.

A reverse transcription PCR (RT-PCR) was carried out on the RNA using the mix iScript Reverse Transcription Supermix (Biorad) and the following thermocycler program: 5 min at 25 °C, 30 min at 42 °C and 5 min at 85 °C. The cDNA was quantified using Qubit 2.0 fluorometer (ssDNA assay kit, Invitrogen, Life Technologies).

Archaeal hyper variable region V4-V5 of the 16S rRNA gene was amplified on the cDNA according to the protocol described by Madigou et al. (Madigou et al., 2018).

16S rRNA tags reads were imported in FROGS. FROGS (Find Rapidly OTU with Galaxy Solution) is a galaxy/CLI workflow designed to produce an OTU count matrix from high depth sequencing amplicon data (Escudié et al., 2018). FROGS abundance file was examined using R CRAN software (version 3.4.4). Considering the dispersion in the total number of reads identified in each sample, archaeal OTUs abundances were normalized with total sum scaling. Only OTUs that exceeded 1% in at least one sample have been taken into account for the analysis.

3. Results and discussion

3.1. Physico-chemical characteristics of the substrates and inoculum

The physico-chemical characteristics of the different substrates and the inoculum are summarised in Table 1. The C/N ratio of fish was lower than the C/N ratio of sludge and grass which were similar. Two key information can be drawn from these analyses. The first one is the higher amount of nitrogen (N) in fish waste (9.5%) compared to sludge and grass (2.46 and 2.2% respectively), explaining the low C/N ratio. This result implies a higher potential to produce ammonia during the fish degradation compared to sludge and grass. The second information is the lower quantity of dissolved organic carbon in sludge (1250 mgC/L) compared to fish

(7921 mgC/L) and grass (7692 mgC/L). It suggests that even if the C/N ratio was similar between grass and sludge, the CH_4 production could start earlier in fish and grass because more readily available carbon was present.

A BMP test was carried out to determine the maximal CH_4 production potential for the different substrates. The kinetics production parameters determined after modelling of the data with Gompertz equation are presented in the Table 2. Degradation started immediately for all the substrates as expected according to the substrate/inoculum ratio used. Sludge-BMP degradation was the slowest (2.09 mgC/D/gCOD) and the less important (44.35 mgC/gCOD of CH_4 was produced). The slow degradation of the sludge was in accordance with the lower DOC initial value observed for the sludge. Fish-BMP and Grass-BMP degradation produced almost the same final amount of CH_4 (circa 119 mgC/gCOD) and at a similar rate (circa 8 mgC/D/gCOD).

3.2. Mono-digestion of the substrates

The specific methane production of wastewater sludge (S-100), fish waste (F-100) and grass (G-100) mono-digestion are presented in the Fig. 1 and the kinetics parameters of production determined after modelling with Gompertz equation are presented in the Table 2. Between 30 and 50 % of the initial quantity was transformed into biogas. Wastewater sludge in the experimental batch mono-digestion produced the lowest final CH_4 quantity (46.62 mgC/gCOD) despite a similar COD amount fed to the bioreactor at the beginning of the experiment. The highest final CH_4 production was observed for fish (F-100, 87.55 mgC/gCOD, compared to respectively 68.43 and 46.62 mgC/gCOD for G-100 and S-100) while the production began earlier and was faster when grass was used as a single substrate (G-100). The faster CH_4 production rate estimated by Gompertz modelling for G-100 can be explained by the higher amount of DOC present in the grass (Supplementary Fig. B.1-A). Because dissolved organic carbon was readily available, the CH_4 production could start earlier and be faster. It is interesting to notice that the kinetic of CH_4 production differed between fish and grass during mono-digestion experiment while they were similar during the

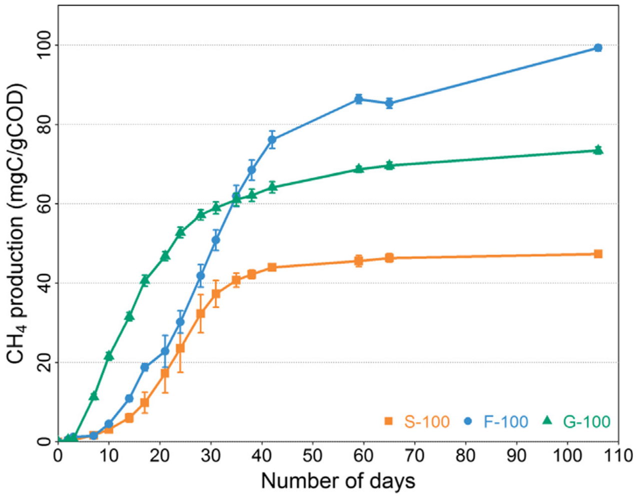

Fig. 1. Cumulated CH_4 production (mgC/gCOD) over time (days) for the different substrates in mono-digestion experiments. Mean values of the triplicate bioreactors for CH_4 productions, error bars represent standard deviation within triplicates. S, F and G stand for Sludge, Fish and Grass respectively.

BMP test. Because the only difference between the mono-digestion experiment and the BMP test was the Substrate/Inoculum ratio, respectively 12/1.2 and 0.7/7 gCOD, it can be hypothesised that the concentration of the methanogenic biomass at the beginning of the experiments played a non-negligible role in the kinetics of the CH_4 production. This assessment was already described in several publications (Hobbs et al., 2018; Zhou et al., 2011).

Biogas productions observed in our experiment was compared to the values described in the literature for similar substrates. Abendroth et al. observed a higher performance of methane production from WAS mono-digestion (250–300 mL/gCOD) (Abendroth et al., 2017) than in our study (95 mL/gCOD). This difference can be explained by the difference of WAS quality according to the preprocess. Indeed it was already described that industrial digesters treating WAS present different performances

Table 2
Kinetics parameters for CH_4 production using Gompertz model for the different mixtures of substrates, BMP tests and the biodegradation indicators. The data are the mean values for the triplicate bioreactors, standard deviations are indicated. μ correspond to the CH_4 production rate, λ to the latency and A to the maximum production.

	Name	μ (mgC/day/gCOD)	λ (day)	A (mgC/gCOD)
Experimental Batchs	F-100	2.35 (±0.57)	10.1 (±0.8)	87.55 (±13.00)
	F-75	3.00 (±0.11)	10.8 (±0.6)	86.31 (±4.93)
	F-50	2.49 (±0.35)	7.7 (±0.8)	72.03 (±4.49)
	F-25	2.31 (±0.10)	9.4 (±0.5)	64.65 (±1.46)
	S-100	2.26 (±0.26)	12.8 (±1.3)	46.62 (±0.8)
	G-25	1.93 (±0.10)	4.5 (±0.6)	42.75 (±5.41)
	G-50	2.97 (±0.21)	3.2 (±1.1)	70.80 (±4.72)
	G-75	3.45 (±0.46)	2.6 (±0.7)	79.30 (±6.99)
	G-100	2.81 (±0.12)	3.1 (±0.2)	68.43 (±0.97)
Mono-Digestion Indicator	F75-MDI	2.33 (±0.49)	10.8 (±0.9)	77.32 (±9.95)
	F50-MDI	2.31 (±0.43)	11.5 (±1.1)	67.09 (±6.90)
	F25-MDI	2.28 (±0.35)	12.1 (±0.5)	56.85 (±3.85)
	G25-MDI	2.40 (±0.24)	10.4 (±1.0)	52.07 (±0.84)
	G50-MDI	2.54 (±0.20)	8.0 (±0.8)	57.53 (±0.89)
	G75-MDI	2.67 (±0.16)	5.5 (±0.5)	62.98 (±0.93)
BMP test	Fish-BMP	8.18 (±0.24)	0	119.35 (±15.28)
	Sludge-BMP	2.09 (±0.17)	0	44.35 (±18.3)
	Grass-BMP	8.47 (±3.67)	0	118.33 (±12.9)
Optimal Digestion Indicator	F75-ODI	6.66 (±0.22)	0	100.60 (±16.04)
	F50-ODI	5.14 (±0.21)	0	81.85 (±16.79)
	F25-ODI	3.61 (±0.19)	0	63.10 (±17.55)
	G25-ODI	6.88 (±2.80)	0	99.84 (±14.25)
	G50-ODI	5.28 (±1.92)	0	81.34 (±15.60)
	G75-ODI	3.69 (±1.05)	0	62.85 (±16.95)

(Rivière et al., 2009; Sundberg et al., 2013). In the same way grass anaerobic digestion performances will greatly depends of its type, treatment or freshness (Prochnow et al., 2009). Fish mono-digestion methane performances will also depends of the type and the part of fish digested (Donoso-Bravo et al., 2015). However in our study the final methane production (198 mL/gCOD) was comparable to the methane performances obtained by Donoso-Bravo et al. which was around 200–300 mL/gCOD.

The concentration of acetic and propionic acids for all batchs are presented in the Supplementary Fig. B.1-B. The acetate maximum accumulation during sludge mono-digestion S-100 was the lowest (47 mgC/L/gCOD) and acetate took around 40 days to be degraded. In comparison, fish and grass mono-digestion (F-100 and G-100) degradation produced a similar level of acetate in 7 days (circa 90 mgC/L/gCOD) suggesting that organic carbon in fish and grass was more readily degradable. However the total acetate degradation occurred in 13 days for G-100 and in 43 days for F-100. These results coupled to the difference in the final methane production between G-100 and F-100 indicated a lower degradability across time of the grass compared to the fish. No butyrate was produced during the sludge degradation while butyrate accumulation was mainly observed in F-100 (maximum 30 mgC/L/gCOD compared to 10 mgC/L/gCOD in G-100). The propionate maximum accumulation was more important for F-100 (47 mgC/L/gCOD) compared to G-100 (37 mgC/L/gCOD). For G-100, the degradation of the propionate started after all the acetate had been totally degraded. In F-100 the degradation was not completed at day 72. An increase of 15 mgC/gCOD of CH_4 was observed between day 70 and the end of the experiment suggesting that a part of the 25 mgC/gCOD of propionate present at day 70, started to be degraded after day 70. Propionate is one of the most important precursors in methane production after acetate (Lawrence and McCarty, 1969) but it is also reported to accumulate easily and cause process inhibition in some cases (Gallert and Winter, 2008; Wang et al., 2009). Anaerobic oxidation of propionic acid is thermodynamically unfavorable and depends on acetate and H_2 content (Boone and Bryant, 1980; Mawson et al., 1991). It is only performed by specific microorganisms. The major pathway for the anaerobic propionate degradation is a syntrophic degradation of propionate linked to H_2 transfer via a methanogen (Ariesyady et al., 2007). Delays observed in the degradation of the propionate for fish mono-digestion F-100 could be explained by the time needed by the appropriate microorganism to grow and by the syntrophy to take place.

The evolution of NH_4^+, pH and NH_3 values during the substrates degradation are presented in the Supplementary Fig. B.1-C. F-100 produced in 7 days around 1300 mg/L of NH_4^+ compared with 350 mg/L for G-100 and S-100 corresponding to a respectively amount of 140 and 30 mg/L of NH_3. Ammonia nitrogen is known to be an inhibitor of the AD, especially free ammonia (NH_3) (Fotidis et al., 2013; Rajagopal et al., 2013). However a wide range of half inhibitory concentrations has been reported between 1.7 and 19 g/L of NH_4^+ (Chen et al., 2008; Poirier et al., 2016) and 50–1400 mgNH_3/L (Rajagopal et al., 2013) depending on multiple factors such as the microbial community, temperature... The highest free ammonia accumulation observed for F-100 was 140 mg/L at day 50. The amount of ammonia observed in F-100 was under the inhibitory values described in the literature. It cannot be excluded that the microbial community was partly inhibited, particularly the methanogens and the propionate degrading acetogenic bacteria which are known to be sensitive to free ammonia (Calli et al., 2005; Westerholm et al., 2011).

3.3. Co-digestion of the substrates

3.3.1. Performances of wastewater sludge co-digestion with fish waste

Evolution of the cumulated CH_4 production over time for the different mixtures is presented in Fig. 2 and the Table 2 details the results of the Gompertz modelling for each mixture. In all cases

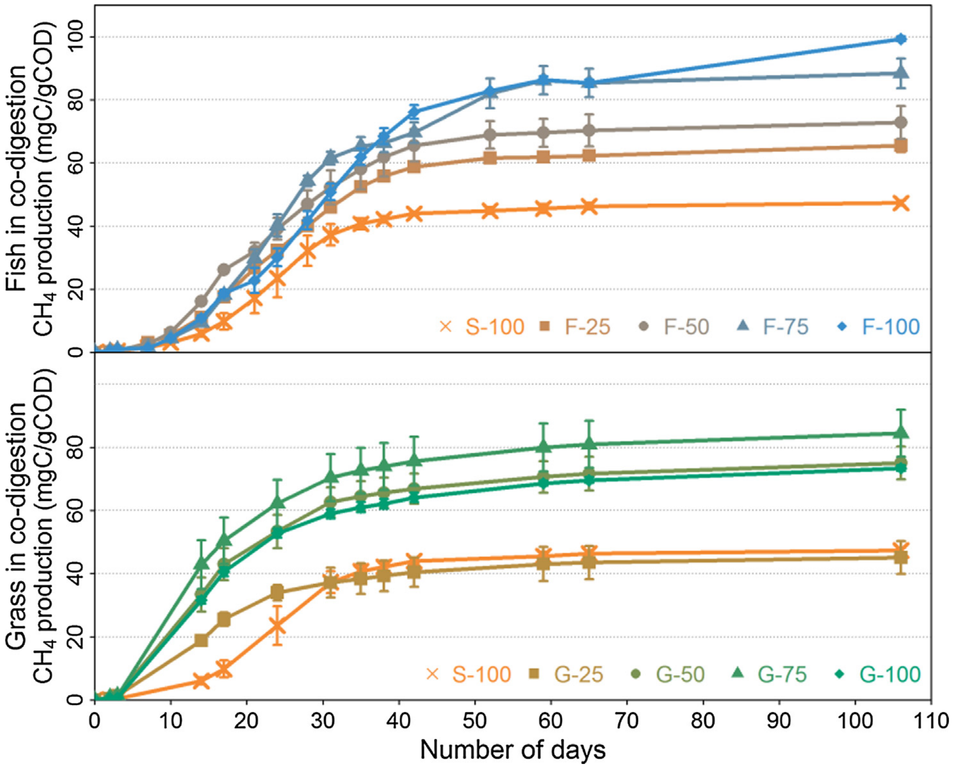

Fig. 2. Cumulated CH_4 production (mgC/gCOD) over time (days) for Fish and Grass used as co-substrates in co-digestion with wastewater sludge. Mean values of the triplicate bioreactors, error bars represent standard deviation within triplicates. S-100 stands for wastewater sludge alone, F-25, F-50, F-75, F-100 stands for respectively 25, 50, 75 or 100% of fish (F) in co-digestion with sludge, G-25, G-50, G-75, G-100 stands for respectively 25, 50, 75 or 100% of Grass (G) in co-digestion with sludge.

the addition of fish enabled to increase the final CH_4 production compared to S-100. For example, F-25, which contains 25% of fish and 75% of sludge, produced 65.58 mgC/gCOD of CH_4, *ie*. 18 mgC/gCOD more than S-100. According to the Gompertz modelling the latency before CH_4 production start was not significantly modified between the different mixtures, but the production rate was increased from 2.31 to 3.00 mgC/D/gCOD for F-25 and F-75 respectively. Dissolved organic carbon accumulation (Supplementary Figure C.1-A) between days 0 to 7, representative of the solid carbon degradation during the early hydrolytic phase, increased when more than 25% (gCOD) of fish was mixed to WAS. This could be explained by the presence of a higher quantity of easily degradable carbon or by a hydrolysis step faster when fish was present than for S-100.

Volatile fatty acids accumulation, presented in the Supplementary Figure C.1-B shows a similar VFA pattern evolution for the mixtures F-25 and F-50 than for S-100. The acetate accumulation profile in F-75 was also similar to S-100 while the propionate maximum accumulation was closed to F-100 (45 mgC/L/gCOD). The consumption of the propionate in F-75 was completely achieved after day 60 while in F-100 the propionate degradation had not started yet. Regarding the butyrate production no significant effect of AcoD was observed. The ammonia accumulation presented in the Supplementary Fig. C.1-C shows an increase of the NH_4^+ level proportional to the amount of fish added in the feeding, indicating that ammonia production was mainly due to fish. Use wastewater sludge to dilute fish waste decreased the NH_4^+ level which seemed to reduce the inhibition of the propionate degrading population observed on the fish mono-digestion. This allowed to increase the propionate degradation rate and to produce the CH_4 faster.

3.3.2. Performances of wastewater sludge co-digestion with garden-grass

In the case of co-digestion with grass, the final CH_4 production was increased only when more than 50% of grass was mixed with sludge compared to sludge mono-digestion (Fig. 2). G-25 presented an earlier CH_4 production but a final production similar to S-100. The more the proportion of grass was important the more the latency to produce CH_4 was reduced (from 4.5 days to 2.6 for G-25 and G-75 respectively). Mixing grass and sludge increased the amount of dissolved organic carbon accumulated during the 7 first days. All the mixtures presented the same evolution of acetate accumulation than G-100 with a higher and faster accumulation than in S-100. However, propionate final accumulation was 2 times higher for the mixtures than for S-100 (20 mgC/L/gCOD) and G-100 (37 mgC/L/gCOD) but propionate degradation for all bioreactors was achieved in 30 days. For all bioreactors of grass co-digestion, the ammonia level stayed stable along the experiment closed to 350 mg/L such as the level in S-100 and G-100.

3.4. Influence of the co-digestion on the methanogenic pathways

Measurement of the biogas carbon stable isotopic composition allowed to follow the evolution of the methanogenic pathway across time (Conrad, 2005). The results are presented in the Fig. 3.

The methane production in S-100 was carried out during the first 20 days mostly by the hydrogenotrophic pathway (α_{app} superior to 1.065). After 20 days, the methanogenic pathway changed progressively from hydrogenotrophic to acetoclastic pathway (α_{app} inferior than 1.055 after 30 days).

In the fish mono-digestion the gas production at the beginning of the experiment was dominated by the acetoclastic pathway. During the first week of the experiment, the methanogenic pathway increased from 1.04 to 1.06, namely from acetoclastic pathway to a mix of methanogenic pathways. This can be explained by a high hydrolytic activity producing H_2 and CO_2 and favouring the gas production by the hydrogenotrophic pathway. During the next 20 days the α_{app} decreased due to the consumption of the acetate by the acetoclastic methanogens. Finally, when all the acetate was degraded,

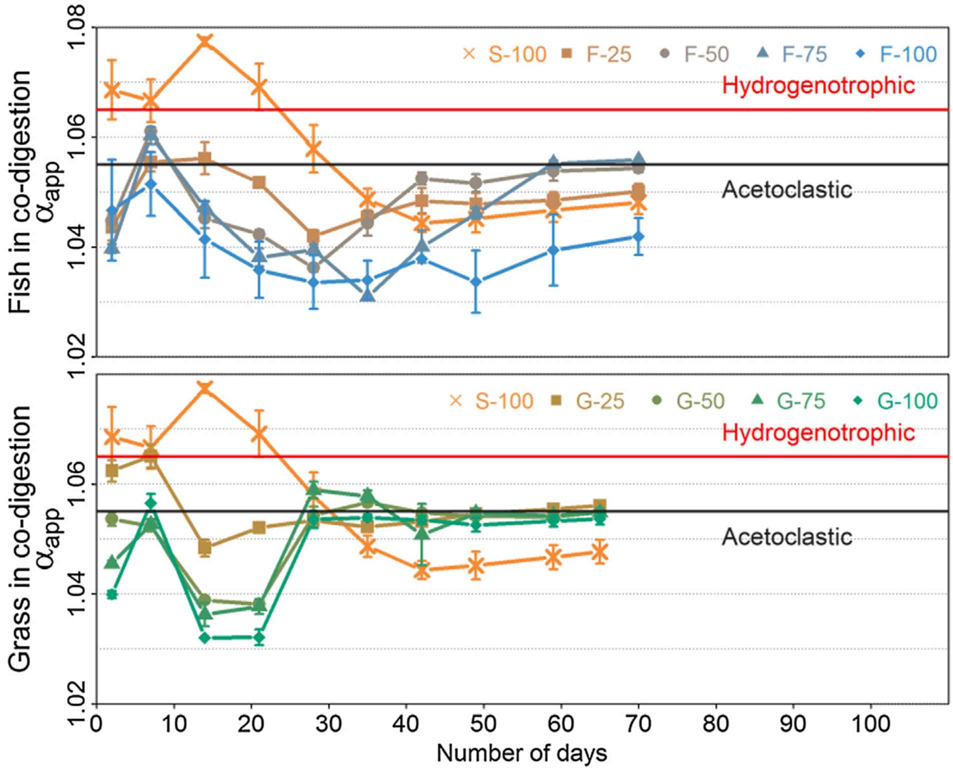

Fig. 3. Apparent isotope fractionation over time (days) for Fish and Grass used as co-substrates in co-digestion with wastewater sludge. Mean values of the triplicate bioreactors, error bars represent standard deviation within triplicates. S-100 stands for wastewater sludge alone, F-25, F-50, F-75, F-100 stands for respectively 25, 50, 75 or 100% of fish (F) in co-digestion with sludge, G-25, G-50, G-75, G-100 stands for respectively 25, 50, 75 or 100% of Grass (G) in co-digestion with sludge.

the α_{app} increased again due to the syntrophic oxidation of the propionate during which methane was produced by acetoclastic and hydrogenotrophic pathways. The evolution across time of the methanogenic pathways for the mixtures of the co-digestion with fish followed the same evolution than F-100 but with the values of the α_{app} corresponding to an intermediate between F-100 and S-100. Because the propionate was not degraded at the same time for the different fish mixtures, the α_{app} evolved differently between the different mixtures. This study shows that addition of fish waste influenced strongly the methanogenic pathway during the co-digestion.

The mono-digestion of grass presented a similar evolution across time of the methanogenic pathway. The beginning of the experiment started in the acetoclastic pathway. After a first increase followed by a decrease of the α_{app} induced by the production of CO_2 then the consumption of the acetate, the α_{app} stabilised at 1.055 due to the propionate degradation. Contrary to the fish mixtures, the co-digestion mixtures of grass presented a distinction between two groups. The methanogenic pathway evolution for G-25 was closer to S-100 with a beginning in the hydrogenotrophic pathway while the methanogenic pathways evolution of G-50 and G-75 was similar to G-100. This differentiation in the methanogenic pathways for the grass mixtures can explain the difference observed in the methane production.

In support of the isotopic fractionation results the active archaeal community was analysed using 16S RNA sequencing during the methane production phase (Fig. 4). *Methanosarcina* genus was the most abundant archaea in all the digesters independently of the feeding composition. This archaea has a versatile methanogenesis metabolism but in regards with the isotopic results the acetoclastic pathway seemed to be dominant. Indeed, except for sludge mono-digestion, the methane was mostly produced by the acetoclastic pathway since the beginning of the experiments. Other hydrogenotrophic archaea were found in the digesters. *Methanoculleus* and *Methanobacterium* genera were found in digesters fed with sludge and/or fish. Their abundances were higher in sludge mono-digestion at the beginning of the methane production (day 14) than in fish fed digesters. This result is in accordance with the isotopic fractionation which showed that hydrogenotrophic pathway was dominant in sludge mono-digestion during the 20th first days. *Methanospirullum* were relatively abundant in digesters fed with grass and sludge, reaching up to 25% in G25 bioreactors. Archaea of *Methanofollis* and *Methanocorpusculum* were found specifically in digesters fed with more than 25 of grass but they remained minority.

3.5. Estimation of the optimal blend of the co-digestion using indicators

In order to evaluate the benefit of the co-digestion, the experimental values of the CH_4 production measured for the bioreactors were compared to two indicators that we built. The first one is the

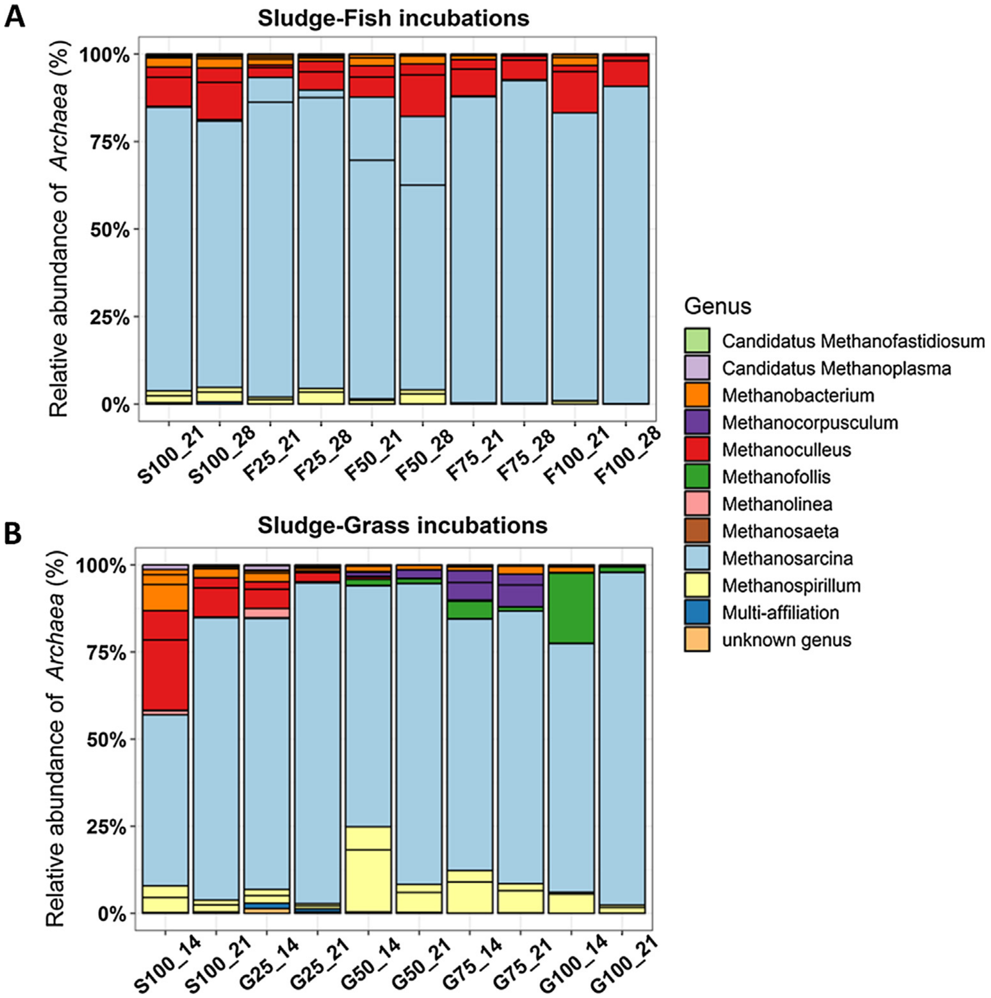

Fig. 4. Taxonomic composition at genus level based on the 16S archaea-specific amplicon sequences. (A) Samples from reactors fed with sludge and/or fish at days 21 and 28 of the experiment (B) Samples from reactors fed with sludge and/or grass at days 14 and 21 of the experiment. Days were selected to correspond to the methane production phase. S100 stands for wastewater sludge alone, F25, F50, F75, F100 stands for respectively 25, 50, 75 or 100% of fish (F) in co-digestion with sludge, G25, G50, G75, G100 stands for respectively 25, 50, 75 or 100% of Grass (G) in co-digestion with sludge.

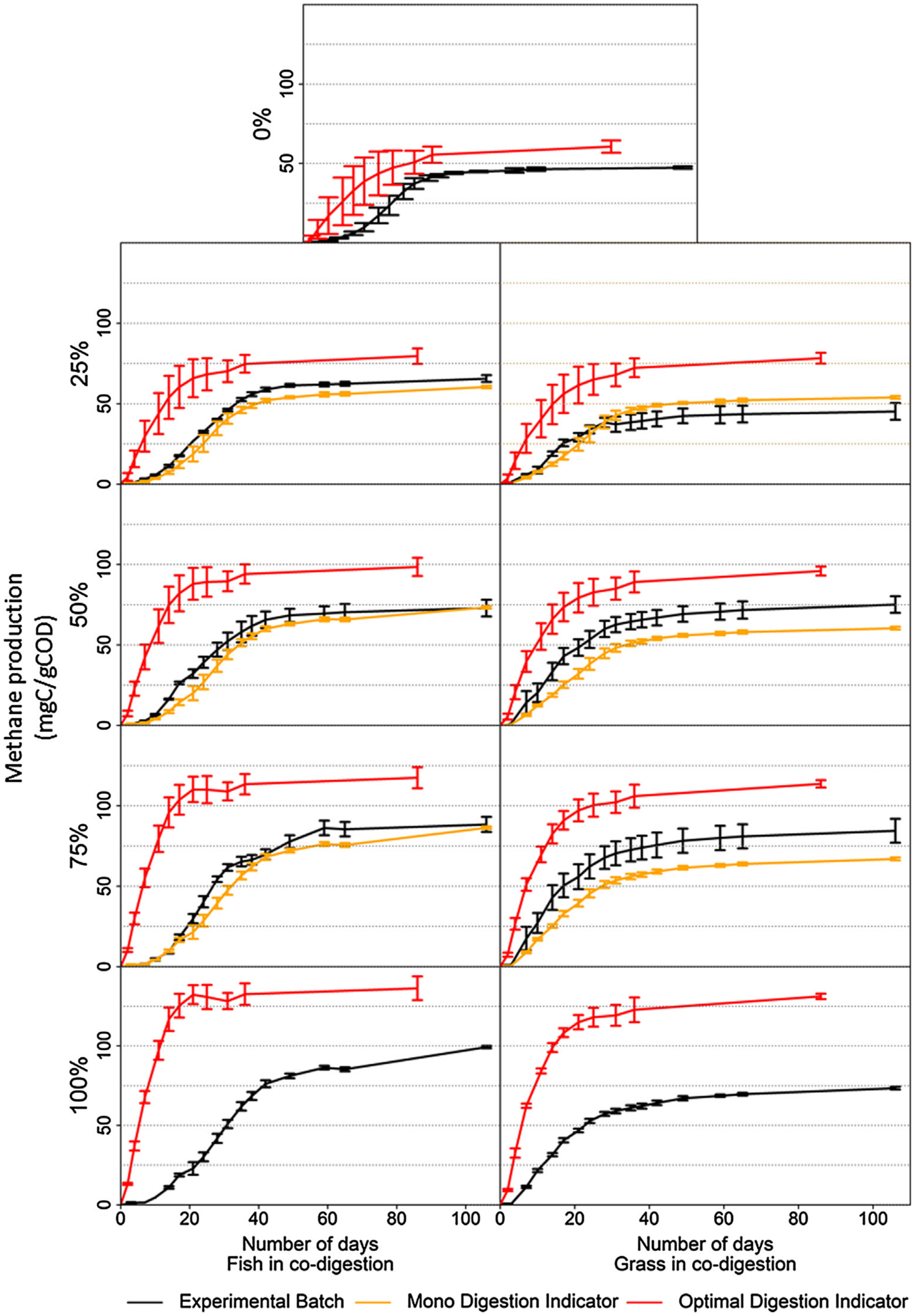

Fig. 5. Experimental Batch methane production (mgC/gCOD) compared to the Maximal Digestion Indicator and the Optimal Digestion Indicator over time (days) for fish and grass substrates in co-digestion with wastewater sludge. Mean values of the triplicate bioreactors for CH_4 productions, error bars represent standard deviation within triplicates. S-100 stands for wastewater sludge alone, F-25, F-50, F-75, F-100 stands for respectively 25, 50, 75 or 100% of fish (F) in co-digestion with sludge, G-25, G-50, G-75, G-100 stands for respectively 25, 50, 75 or 100% of Grass (G) in co-digestion with sludge.

empirical maximum production that could be obtained under optimal conditions (determined with BMP) and called thereafter Optimal Digestion Indicator (ODI). For the different mix, ODI was calculated as a linear combination of the BMP experimental values obtained for Sludge-BMP, Fish-BMP and Grass-BMP using the percentage of each co-substrate as coefficient. The second indicator is the empirical production that could be obtained under experimental conditions and called Mono-Digestion Indicator (MDI). MDI was calculated in the same way than ODI but using the linear combination of the mono-digestion experimental values. Fig. 5 represents the comparison of the experimental CH_4 production profiles to the indicators for each mixture. The Table 2 summarises the kinetics production parameters determined after modelling the data with Gompertz equation for all the experiments and indicators.

The comparison of the experimental mono-digestion (F-100, G-100 and S-100) to the Optimal Digestion Indicator was already

described in details in the section dedicated to the mono-digestion. The ODI was higher than the experimental methane production but the co-digestion with fish or grass allowed to get close to the final methane production and/or the production rate of the mixtures to the ODI.

For the co-digestion with fish waste the comparison showed no significant differences between the experimental final methane production and the MDI. However the methane production rate for the F-75 (3.00 mgC/days/gCOD) was increased comparing to the MDI (2.33 mgC/days/gCOD). This result, taken with the chemical results (propionate) and the methanogenic pathway analysis, indicates that use of 75% of fish seemed to be the optimal proportion to enhance the methane production from sewage sludge. Indeed, the propionate was consumed earlier and was associated to a faster return to the acetoclastic methanogenic pathway. On an industrial point of view if the aim is to enhance the final CH_4 production from wastewater sludge, fish waste is a good candidate even at a low quantity. The other advantage to treat fish waste in co-digestion is to limit the risk of an inhibition by the ammonia and the propionate which are accumulated during the degradation in mono-digestion. The high potential of VFA production from fish was already studied (Bermúdez-Penabad et al., 2017). Even if no such inhibition was observed in our system, the potentiality of ammonia and VFA accumulation was observed. However, in case of CH_4 production enhancement, the VFA production can inhibit the system and the co-digestion can be a solution to overcome this inhibition (Xu et al., 2017).

Regarding the co-digestion with grass, G-25 maximal methane production was slightly lower than the one estimated by the MDI. In contrast, G-50 and G-75 allowed to increase the maximal production by 1.2 times and the CH_4 production rate from 1.2 and 1.3 respectively. Because the experimental CH_4 production was higher than the MDI prediction during all the experiment for G-50 and G-75, it can be supposed that a synergistic effect occurred between grass and wastewater sludge at these proportions. The mixture improving the AD performances the more in term of CH_4 production is G-75. The substrate degradation started earlier, the methane production rate was improved by 1.2 times and the maximal production was increased by 1.7 times compared to the mono-digestion of wastewater of sludge and 0.9 compared to the mono-digestion of grass.

4. Conclusion

Increasing the fish concentration in co-digestion with wastewater sludge gradually improved the final methane production up to 1.9 times when 75% of fish was added. On the contrary grass enabled to improve the final methane production from WAS only when more than 25% of grass was added. Adding more than 50% of grass improved both the production rate and the final production by to 1.5 and 1.7 times respectively. Specifically built indicators showed that using 75% of fish or grass as co-substrate with sewage sludge enabled to obtain the maximum final methane production. In nearly all the bioreactors, archaea from *Methanosarcina* genus accounted for more than 75% of the archaeal diversity. No significant difference in the methanogenic pathways was observed across time between fish and grass mono-digestion. It was mostly acetoclastic while wastewater sludge mono-digestion changed from hydrogenotrophic to acetoclastic methanogenesis pathway. The anaerobic co-digestion allowed to limit the variation between the methanogenesis pathway of the sludge.

Declarations of interest

None.

Acknowledgements

We want to thank Lénaïck Rouillac and Nadine Derlet from the Irstea PROSE analytical division for their technical support. Our acknowledgments also go to SUEZ Environment for providing us access to the wastewater treatment plant of Valenton.

Funding

This work was supported by the National Research Agency (ANR-16-CE05-0014). The funders had no role in study design, data collection and analysis, decision to publish, or preparation of the manuscript.

Appendix A. Supplementary material

Supplementary data to this article can be found online at https://doi.org/10.1016/j.wasman.2019.03.016.

References

Abendroth, C., Simeonov, C., Peretó, J., Antúnez, O., Gavidia, R., Luschnig, O., Porcar, M., 2017. From grass to gas: Microbiome dynamics of grass biomass acidification under mesophilic and thermophilic temperatures. Biotechnol. Biofuels 10, 1–12. https://doi.org/10.1186/s13068-017-0859-0.

Anthonisen, A., Loehr, R., Prakasam, T., Srinath, E., 1976. Inhibition of nitrification by ammonia and nitrous acid. J. Water Pollut. Control Fed. 48, 835–852. https://doi.org/10.1017/CBO9781107415324.004.

Ariesyady, H.D., Ito, T., Okabe, S., 2007. Functional bacterial and archaeal community structures of major trophic groups in a full-scale anaerobic sludge digester. Water Res. 41, 1554–1568. https://doi.org/10.1016/j.watres.2006.12.036.

Astals, S., Esteban-Gutiérrez, M., Fernández-Arévalo, T., Aymerich, E., García-Heras, J.L., Mata-Alvarez, J., 2013. Anaerobic digestion of seven different sewage sludges: a biodegradability and modelling study. Water Res. 47, 6033–6043. https://doi.org/10.1016/j.watres.2013.07.019.

Astals, S., Venegas, C., Peces, M., Jofre, J., Lucena, F., Mata-Alvarez, J., 2012. Balancing hygienization and anaerobic digestion of raw sewage sludge. Water Res. 46, 6218–6227. https://doi.org/10.1016/j.watres.2012.07.035.

Bermúdez-Penabad, N., Kennes, C., Veiga, M.C., 2017. Anaerobic digestion of tuna waste for the production of volatile fatty acids. Waste Manage. 68, 96–102. https://doi.org/10.1016/j.wasman.2017.06.010.

Boone, D.R., Bryant, M.P., 1980. Propionate-Degrading Bacterium, Syntrophobacter wolinii sp. nov. gen. nov., from Methanogenic. Ecosystems 40, 626–632.

Borowski, S., Kubacki, P., 2015. Co-digestion of pig slaughterhouse waste with sewage sludge. Waste Manage. 40, 119–126. https://doi.org/10.1016/j.wasman.2015.03.021.

Brand, W.A., 1996. High precision isotope ratio monitoring techniques in mass spectrometry. J. Mass Spectrom. 31, 225–235.

Calli, B., Mertoglu, B., Inanc, B., Yenigun, O., 2005. Effects of high free ammonia concentrations on the performances of anaerobic bioreactors. Process Biochem. 40, 1285–1292. https://doi.org/10.1016/j.procbio.2004.05.008.

Chapleur, O., Bize, A., Serain, T., Mazéas, L., Bouchez, T., 2014. Co-inoculating ruminal content neither provides active hydrolytic microbes nor improves methanization of 13C-cellulose in batch digesters. FEMS Microbiol. Ecol. 87, 616–629. https://doi.org/10.1111/1574-6941.12249.

Chen, Y., Cheng, J.J., Creamer, K.S., 2008. Inhibition of anaerobic digestion process: a review. Bioresour. Technol. 99, 4044–4064.

Conrad, R., 2005. Quantification of methanogenic pathways using stable carbon isotopic signatures: a review and a proposal. Org. Geochem. 36, 739–752. https://doi.org/10.1016/j.orggeochem.2004.09.006.

Dai, X., Li, X., Zhang, D., Chen, Y., Dai, L., 2016. Simultaneous enhancement of methane production and methane content in biogas from waste activated sludge and perennial ryegrass anaerobic co-digestion: The effects of pH and C/N ratio. Bioresour. Technol. 216, 323–330. https://doi.org/10.1016/j.biortech.2016.05.100.

Donoso-Bravo, A., Bindels, F., Gerin, P.A., Vande Wouwer, A., 2015. Anaerobic biodegradability of fish remains: Experimental investigation and parameter estimation. Water Sci. Technol. 71, 922–928. https://doi.org/10.2166/wst.2015.047.

Eiroa, M., Costa, J.C., Alves, M.M., Kennes, C., Veiga, M.C., 2012. Evaluation of the biomethane potential of solid fish waste. Waste Manage. 32, 1347–1352. https://doi.org/10.1016/j.wasman.2012.03.020.

Escudié, F., Auer, L., Bernard, M., Mariadassou, M., Cauquil, L., Vidal, K., Maman, S., Hernandez-Raquet, G., Combes, S., Pascal, G., 2018. FROGS: Find, rapidly, OTUs with galaxy solution. Bioinformatics 34, 1287–1294.

FAO, 2016. The state of world fisheries and aquaculture 2016. Rome. https://doi.org/10.5860/CHOICE.50-5350.

Fotidis, I.A., Karakashev, D., Kotsopoulos, T.A., Martzopoulos, G.G., Angelidaki, I., 2013. Effect of ammonium and acetate on methanogenic pathway and methanogenic community composition. FEMS Microbiol. Ecol. 83, 38–48. https://doi.org/10.1111/j.1574-6941.2012.01456.x.

Gallert, C., Winter, J., 2008. Propionic acid accumulation and degradation during restart of a full-scale anaerobic biowaste digester. Bioresour. Technol. 99, 170–178. https://doi.org/10.1016/j.biortech.2006.11.014.

Hansen, K.H., Angelidaki, I., Ahring, B.K., 1998. Anaerobic digestion of swine manure: inhibition by ammonia. Water Res. 32, 5–12. https://doi.org/10.1016/S0043-1354(97)00201-7.

Hidaka, T., Arai, S., Okamoto, S., Uchida, T., 2013. Anaerobic co-digestion of sewage sludge with shredded grass from public green spaces. Bioresour. Technol. 130, 667–672. https://doi.org/10.1016/j.biortech.2012.12.068.

Hobbs, S.R., Landis, A.E., Rittmann, B.E., Young, M.N., Parameswaran, P., 2018. Enhancing anaerobic digestion of food waste through biochemical methane potential assays at different substrate: inoculum ratios. Waste Manage. 71, 612–617. https://doi.org/10.1016/j.wasman.2017.06.029.

Lawrence, A.W., McCarty, P.L., 1969. Kinetics of methane fermentation in anaerobic treatment. J. Water Pollut. Control Fed. 41, 1–17. https://doi.org/10.2307/25036255.

Luostarinen, S., Luste, S., Sillanpää, M., 2009. Increased biogas production at wastewater treatment plants through co-digestion of sewage sludge with grease trap sludge from a meat processing plant. Bioresour. Technol. 100, 79–85. https://doi.org/10.1016/j.biortech.2008.06.029.

Madigou, C., Lê Cao, K.-A., Bureau, C., Mazéas, L., Déjean, S., Chapleur, O., 2018. Ecological consequences of abrupt temperature changes in anaerobic digesters. Chem. Eng. J. 361, 266–277. https://doi.org/10.1016/J.CEJ.2018.12.003.

Mata-Alvarez, J., Dosta, J., Romero-Güiza, M.S., Fonoll, X., Peces, M., Astals, S., 2014. A critical review on anaerobic co-digestion achievements between 2010 and 2013. Renew. Sustain. Energy Rev. 36, 412–427. https://doi.org/10.1016/j.rser.2014.04.039.

Mawson, A.J., Earle, R.L., Larsen, V.F., 1991. Degradation of acetic and propionic acids in the methane fermentation. Water Res. 25, 1549–1554. https://doi.org/10.1016/0043-1354(91)90187-U.

Park, K.Y., Jang, H.M., Park, M.-R., Lee, K., Kim, D., Kim, Y.M., 2016. Combination of different substrates to improve anaerobic digestion of sewage sludge in a wastewater treatment plant. Int. Biodeterior. Biodegrad. 109, 73–77. https://doi.org/10.1016/j.ibiod.2016.01.006.

Pitk, P., Kaparaju, P., Palatsi, J., Affes, R., Vilu, R., 2013. Co-digestion of sewage sludge and sterilized solid slaughterhouse waste: Methane production efficiency and process limitations. Bioresour. Technol. 134, 227–232. https://doi.org/10.1016/j.biortech.2013.02.029.

Poirier, S., Desmond-Le Quéméner, E., Madigou, C., Bouchez, T., Chapleur, O., 2016. Anaerobic digestion of biowaste under extreme ammonia concentration: identification of key microbial phylotypes. Bioresour. Technol. 207, 92–101. https://doi.org/10.1016/j.biortech.2016.01.124.

Prabhu, M.S., Mutnuri, S., 2016. Anaerobic co-digestion of sewage sludge and food waste. Waste Manage. Res. 34, 307–315.

Prochnow, A., Heiermann, M., Plöchl, M., Amon, T., Hobbs, P.J., 2009. Bioenergy from permanent grassland – a review: 1. Biogas. Bioresour. Technol. 100, 4945–4954. https://doi.org/10.1016/j.biortech.2009.05.069.

Rajagopal, R., Massé, D.I., Singh, G., 2013. A critical review on inhibition of anaerobic digestion process by excess ammonia. Bioresour. Technol. 143, 632–641. https://doi.org/10.1016/j.biortech.2013.06.030.

Rivière, D., Desvignes, V., Pelletier, E., Chaussonnerie, S., Guermazi, S., Weissenbach, J., Li, T., Camacho, P., Sghir, A., 2009. Towards the definition of a core of microorganisms involved in anaerobic digestion of sludge. ISME J. 3, 700–714. https://doi.org/10.1038/ismej.2009.2.

Sugimoto, A., Hong, X., Wada, E., 1991. Rapid and simple measurement of carbon isotope ratio of bubble methane using GC/C/IRMS. Mass Spectrosc. https://doi.org/10.5702/massspec.39.261.

Sundberg, C., Al-Soud, W.A., Larsson, M., Alm, E., Yekta, S.S., Svensson, B.H., Sørensen, S.J., Karlsson, A., 2013. 454 pyrosequencing analyses of bacterial and Archaeal richness in 21 full-scale biogas digesters. FEMS Microbiol. Ecol. 85, 612–626. https://doi.org/10.1111/1574-6941.12148.

Wang, Y., Zhang, Y., Wang, J., Meng, L., 2009. Effects of volatile fatty acid concentrations on methane yield and methanogenic bacteria. Biomass Bioenergy 33, 848–853. https://doi.org/10.1016/j.biombioe.2009.01.007.

Ward, A.J., Hobbs, P.J., Holliman, P.J., Jones, D.L., 2008. Optimisation of the anaerobic digestion of agricultural resources. Bioresour. Technol. 99, 7928–7940. https://doi.org/10.1016/j.biortech.2008.02.044.

Westerholm, M., Müller, B., Arthurson, V., Schnürer, A., 2011. Changes in the Acetogenic population in a mesophilic anaerobic digester in response to increasing ammonia concentration. Microbes Environ. 26, 347–353. https://doi.org/10.1264/jsme2.ME11123.

Whiticar, M.J., Faber, E., Schoell, M., 1986. Biogenic methane formation in marine and freshwater environments: CO2reduction vs. acetate fermentation-Isotope evidence. Geochim. Cosmochim. Acta 50, 693–709. https://doi.org/10.1016/0016-7037(86)90346-7.

Wickham, R., Galway, B., Bustamante, H., Nghiem, L.D., 2016. Biomethane potential evaluation of co-digestion of sewage sludge and organic wastes. Int. Biodeterior. Biodegradation 113, 3–8. https://doi.org/10.1016/j.ibiod.2016.03.018.

Xu, J., Mustafa, A.M., Sheng, K., 2017. Effects of inoculum to substrate ratio and co-digestion with bagasse on biogas production of fish waste. Environ. Technol. 38, 2517–2522. https://doi.org/10.1080/09593330.2016.1269837.

Zhou, Y., Zhang, Z., Nakamoto, T., Li, Y., Yang, Y., Utsumi, M., Sugiura, N., 2011. Influence of substrate-to-inoculum ratio on the batch anaerobic digestion of bean curd refuse-okara under mesophilic conditions. Biomass Bioenergy 35, 3251–3256. https://doi.org/10.1016/j.biombioe.2011.04.002.
